# Association of Combined Cardiometabolic and Muscle Impairment Index with Cardiovascular-Kidney-Metabolic Syndrome Progression Among Middle-Aged and Older Adults: A Prospective Two-Cohort Study

**DOI:** 10.3390/healthcare14193228

**Published:** 2026-10-01

**Authors:** Zishun Liu, Xiaoying Ren, Chang Su, Guang Wang, Jia Liu

**Affiliations:** Department of Endocrinology, Beijing Chao-Yang Hospital, Capital Medical University, Beijing 100020, China; 19903290126@163.com (Z.L.); rxy178323779@163.com (X.R.); suchang309@163.com (C.S.)

**Keywords:** cardiometabolic index, cardiometabolic index–muscle-loss index, cardiovascular disease, cardiovascular–kidney–metabolic syndrome, muscle-loss index, sarcopenic obesity

## Abstract

**Introduction:** Advanced cardiovascular-kidney-metabolic (CKM) syndrome portends poor cardiovascular outcomes. The cardiometabolic index (CMI) and muscle-loss index (MLI) independently reflect metabolic and cardiovascular risk, but their combined effect on CKM syndrome remains unexplored. We aimed to evaluate the association of CMI-MLI with advanced CKM stages cross-sectionally and with incident CVD longitudinally, among adults aged 45 years and older. **Methods:** We analyzed data from adults aged ≥45 years in the CHARLS (2011–2020) and NHANES (1999–2004). The CMI-MLI index was calculated as the natural logarithm of CMI × MLI, where CMI = (triglycerides/HDL-cholesterol) × (waist/height) and MLI = cystatin C/creatinine. CKM syndrome was staged from 0 to 4 per metabolic, cardiovascular, and renal status, with Stage 3 defined by a high 10-year Framingham risk (≥21.6% in men, ≥21.5% in women) or advanced CKD. Logistic regression assessed the cross-sectional association of CMI-MLI with advanced CKM stages, with a sensitivity analysis defining Stage 3 solely by advanced CKD to address potential circularity; Cox regression evaluated its relationship with incident cardiovascular disease (CVD). **Results**: Among 6921 CHARLS and 3185 NHANES participants (the latter serving as a cross-sectional replication cohort), higher CMI-MLI quartiles were significantly associated with advanced CKM stages in both cohorts (*p* for trend <0.0001). Over a median 9-year follow-up of 5768 CHARLS participants with baseline CKM stages 0–3, 1384 incident CVD events occurred. Participants in the highest CMI-MLI quartile had a significantly higher risk of incident CVD (HR 1.35, 95% CI 1.15–1.59) than those in the lowest quartile, and each SD increase in CMI-MLI was independently associated with a higher CVD risk (HR 1.11, 95% CI 1.04–1.17). **Conclusions**: CMI-MLI was significantly associated with advanced CKM stages and independently predicted incident CVD among individuals free of baseline disease. The cross-sectional association was attenuated when Stage 3 was defined solely by advanced CKD, suggesting partial circularity between the CMI components and the risk score, whereas the prospective association with incident CVD was unaffected. Although its discriminatory ability was modest (area under the curve 0.605) and its incremental value over CMI alone was limited, CMI-MLI may serve as a simple, parsimonious marker for identifying individuals with concurrent cardiometabolic and muscle-related risk.

## 1. Introduction

Globally, cardiovascular disease (CVD) is a major driver of mortality and imposes a substantial disease burden that is expected to rise with population aging [1,2]. The cardiovascular-kidney-metabolic (CKM) syndrome, a novel construct put forward by the American Heart Association (AHA), underscores the intricate interplay among metabolic abnormalities, CVD, and chronic kidney disease (CKD) [3]. With increasing research attention, a well-defined CKM staging system has been established, covering stages 0 (lack of risk factors) to 4 (established cardiovascular disease) [3]. This staging framework demonstrates a gradual increase in CVD risk with advancing CKM stages, underscoring the critical importance of early identification in halting disease progression. The CKM syndrome affects a large proportion of the adult population. In a recent analysis of US adults, the age-standardized prevalence of CKM syndrome was 13.6% for stage 0, 29.9% for stage 1, 43.7% for stage 2, 4.7% for stage 3, and 8.1% for stage 4, with advanced stages (3–4) accounting for 12.8% [4]. Globally, cardiovascular disease caused 18.6 million deaths and 523 million prevalent cases in 2019, with a particularly heavy burden among middle-aged and older adults [1]. Sarcopenic obesity, marked by simultaneous low muscle quantity and obesity, is highly prevalent in older adults and promotes progression toward advanced CKM syndrome [5]. However, traditional cardiovascular risk assessment tools, such as the AHA PREVENT equations and the ASCVD Risk Calculator [6,7], depend largely on conventional risk factors. They often fail to capture the distinctive metabolic and musculoskeletal alterations in older adults with sarcopenic obesity. Therefore, novel composite biomarkers are urgently needed to improve risk stratification for the progression to advanced CKM syndrome in this vulnerable population.

The cardiometabolic index (CMI) was initially proposed by Ichiro and colleagues in 2015 [8]. As a surrogate marker for both central obesity and insulin resistance (IR) [9,10], CMI has been validated by accumulating evidence for its predictive value in metabolic abnormalities and atherosclerotic cardiovascular diseases [11,12,13]. A recent study demonstrated that elevated CMI exhibits a significant correlation with CKM stage progression and acts as a straightforward and practical marker for evaluating CKM-related risk [14]. The serum creatinine-to-cystatin C ratio (Cr/Cys C), identified by Kim et al. as the sarcopenia index (SI), is a well-validated indicator for the diagnosis and prediction of sarcopenia [15,16]. Emerging evidence has linked SI to incident CVD and cardiovascular mortality [17,18], with recent studies demonstrating its value as a biomarker for cardiovascular risk assessment in patients with stage 0–3 CKM syndrome [19]. We therefore constructed a novel muscle-loss index (MLI) as the inverse of SI to serve as a surrogate marker of the severity of muscle impairment.

However, to our knowledge, CMI [8,11,13,14] and the sarcopenia index [15,16,17,18,19] have been examined only as independent risk markers, and recent reviews of sarcopenic obesity and cardiovascular disease [10,20,21,22] have not reported any combined cardiometabolic–muscle index; no study has explored their combined effect on CKM staging or incident CVD. This represents a knowledge gap, particularly for middle-aged and older adults who frequently present with concurrent cardiometabolic and muscle impairment. Adults aged ≥45 years were the focus because this population bears the highest burden of cardiometabolic abnormalities [1,2] and age-related muscle impairment [20,23], and because CHARLS was designed specifically for adults aged 45 years and older [24]. Given that IR, abdominal obesity, and muscle impairment frequently coexist and mutually reinforce one another through shared pathways of chronic inflammation and metabolic dysregulation [25,26], a marker integrating both cardiometabolic and musculoskeletal dimensions may better capture the multidimensional pathophysiology of CKM syndrome than either component alone. We therefore constructed a novel composite biomarker, the cardiometabolic index-muscle-loss index (CMI-MLI), by combining CMI and MLI. In this study, we aimed to evaluate the association of a novel composite index, CMI-MLI, with (i) advanced CKM stages cross-sectionally and (ii) incident CVD longitudinally, among adults aged 45 years and older, using data from CHARLS and NHANES.

## 2. Materials and Methods

### 2.1. Study Design and Population

This research employed data from two nationwide representative surveys: CHARLS (2011–2020) and NHANES (1999–2004 cycles). CHARLS represents a large-scale, nationwide community-based longitudinal study in the Chinese population 45 years of age and older. Launched in 2011 (Wave 1), this nationwide population-based survey spans 150 counties and districts within 28 Chinese provinces. It gathers detailed data regarding demographic features and socioeconomic position, lifestyle habits, and health conditions, and five rounds of in-person follow-ups were completed by 2020 [24]. For the current study, we included information collected in the 2011, 2013, 2015, 2018, and 2020 survey waves (corresponding to Waves 1–5). NHANES represents a large-scale survey aimed at gathering health-related information among the general US population, using a stratified multistage random sampling strategy to ensure national representativeness [27]. Information from the 1999–2004 rounds of the survey was included, since both serum Cr and serum Cys C were available for the calculation of CMI-MLI, whereas other cycles were excluded due to missing Cys C data. As a cross-sectional survey, NHANES does not provide prospective follow-up for incident cardiovascular events; accordingly, the longitudinal analysis was restricted to the CHARLS cohort, and NHANES served solely as a cross-sectional replication cohort.

**Figure 1:**
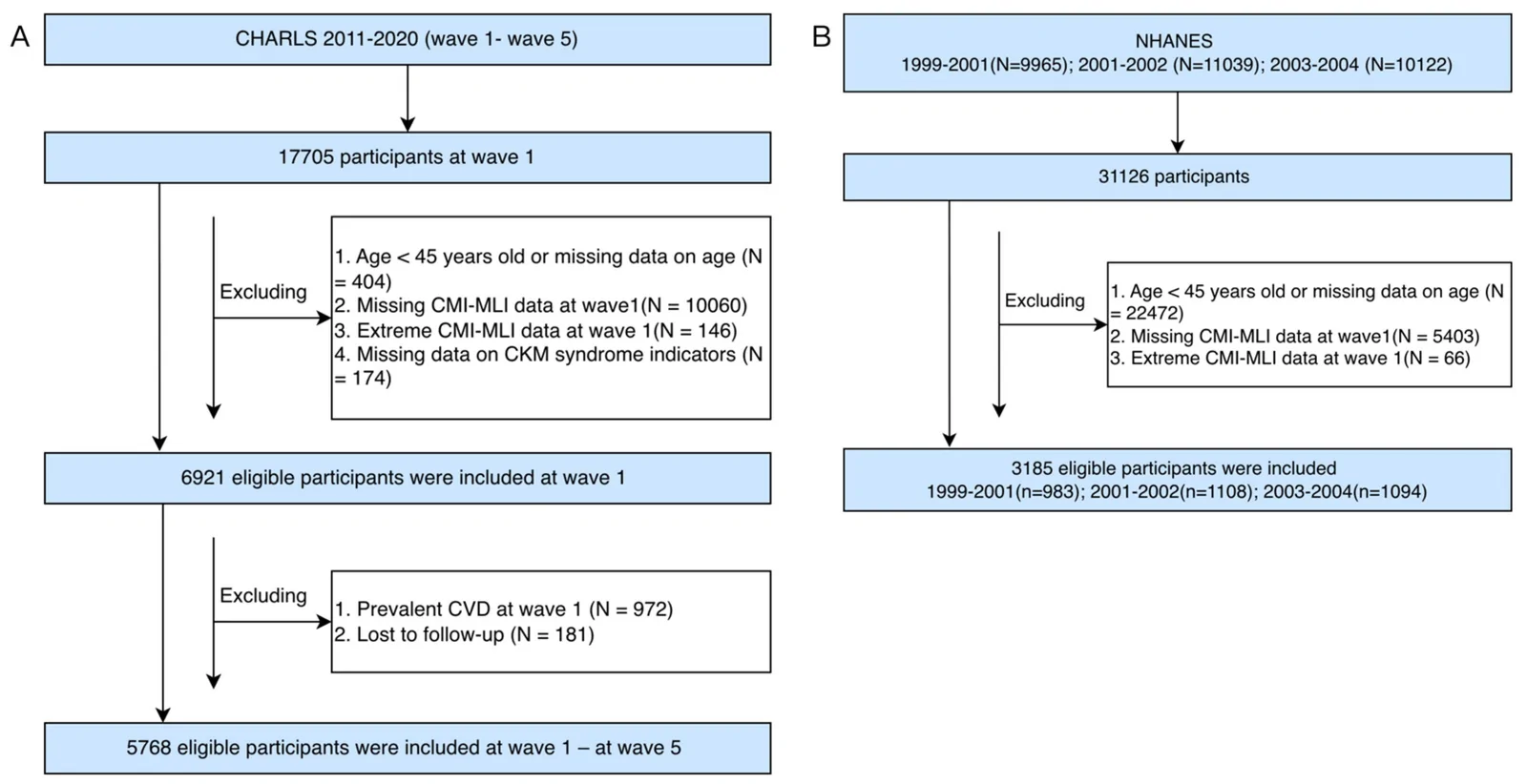
Participant selection flowchart. (**A**) shows the CHARLS cohort and (**B**) shows the NHANES cohort. Abbreviations: NHANES, National Health and Nutrition Examination Survey; CHARLS, China Health and Retirement Longitudinal Study; CMI-MLI: cardiometabolic index-muscle-loss index; CVD, cardiovascular disease; CKM, cardiovascular-kidney-metabolic syndrome.

The inclusion and exclusion criteria for this study were as follows. Adults aged 45 years or older were included. For CHARLS, participants were excluded if they had missing CMI-MLI data (n = 10,060), extreme CMI-MLI values outside the 1st–99th percentile (n = 146), or missing CKM staging components (n = 174), yielding 6921 participants for the cross-sectional analysis. For the longitudinal analysis, we further excluded participants with prevalent CVD at baseline (n = 972) and those lost to follow-up (n = 181), leaving 5768 participants. For NHANES, participants were excluded if they had missing CMI (n = 5195) or MLI (n = 208) data, or extreme CMI-MLI values outside the 1st–99th percentile (n = 66), yielding 3185 participants. The detailed participant selection is shown in Figure 1.

### 2.2. Definition of CMI, MLI, and CMI-MLI

In this research, CMI was calculated as the product of the triglyceride to high-density lipoprotein cholesterol ratio (TG/HDL-C) and the waist-to-height ratio (WHtR) [13]. Specifically, CMI = (TG [mg/dL]/HDL-C [mg/dL]) × (waist [cm]/height [cm]). MLI was calculated as cystatin C (mg/L) divided by creatinine (mg/dL), the reciprocal of the sarcopenia index (SI = creatinine/cystatin C × 100) [17]; the multiplicative scaling factor of 100 was omitted for simplicity, which does not affect the biological interpretation or the statistical associations [28].

Higher MLI values indicate more severe muscle impairment. MLI should be interpreted as a surrogate biochemical marker of muscle impairment rather than a direct diagnostic measure of sarcopenia, because creatinine and cystatin C are influenced by renal function, age, inflammation, and nutrition. Because ln(CMI − MLI) = ln(CMI) + ln(MLI), the index is additive on the logarithmic scale and assigns equal weight to the two components on this scale. This construction follows established composite metabolic indices—such as the lipid accumulation product (LAP), visceral adiposity index (VAI), and triglyceride-glucose index (TyG)—which are likewise expressed as products or ratios of their components and analyzed on the logarithmic scale [29]. In the present study, the raw CMI-MLI was markedly right-skewed (skewness 2.43), and natural logarithmic transformation substantially improved its normality (skewness 0.21, Figure S1), thereby stabilizing variance, improving the linearity of its relationship with outcomes, and attenuating the influence of extreme values. The adequacy of this equal-weight single-index formulation was verified by formal model comparison. CMI-MLI was evaluated both as a continuous measure and as a categorical variable stratified by quartiles. Specific information on the transformation parameters applied during calculation is documented in Table S1.

### 2.3. Definitions of CKM Syndrome Stages

CKM syndrome was stratified into five stages (0–4) according to the American Heart Association framework [3], with detailed classification criteria provided in Table S2. In brief, Stage 0 denotes the absence of CKM risk factors; Stage 1, overweight/obesity, abdominal obesity, or prediabetes without other risk factors; Stage 2, metabolic risk factors (hypertriglyceridemia, hypertension, diabetes, or metabolic syndrome) or moderate-risk CKD [30]; Stage 3, subclinical CVD; and Stage 4, self-reported cardiovascular disease (coronary artery disease, heart failure, or stroke). Because direct measures of subclinical CVD were unavailable, Stage 3 was defined using risk equivalents: in CHARLS, a high 10-year Framingham risk (≥21.6% in men or ≥21.5% in women) or G4/G5 CKD (eGFR < 30 mL/min/1.73 m^2^); in NHANES, a 10-year PREVENT-estimated cardiovascular risk ≥ 20% or very-high-risk CKD. The 10-year risk for CHARLS was estimated using the Framingham risk score for general cardiovascular disease, which incorporates age, sex, total cholesterol, HDL-cholesterol, systolic blood pressure, smoking, and diabetes, consistent with a previous CHARLS-based CKM study [31,32]. Because UACR was unavailable in CHARLS, CKD was staged by eGFR alone. Because the Framingham risk score shares HDL-cholesterol with the CMI component, the cross-sectional association between CMI-MLI and advanced CKM stages may be partly circular; we therefore performed a sensitivity analysis defining Stage 3 solely by G4/G5 CKD. CKM stages 3 and 4 were combined as advanced CKM stages [3].

### 2.4. Definition of Clinical Outcomes

Among the CHARLS and NHANES cohorts, our baseline cross-sectional assessments focused primarily on the association between CMI-MLI values and the risk of advanced CKM syndrome. Within the prospective analysis of CHARLS participants, the primary endpoint was the newly developed CVD events across follow-up waves 2 to 5. In the CHARLS cohort, CVD status was ascertained using two self-reported questionnaire items, in accordance with prior research: (1) Did a licensed physician ever inform you that you had suffered from a stroke? (2) Have you ever received a diagnosis of any heart disorder, including congestive heart failure, coronary heart disease, myocardial infarction, or angina, from a physician? Those who answered “yes” to either inquiry were regarded as having physician-confirmed CVD [33,34]. The duration of follow-up was calculated from the initial assessment to the self-reported onset date of cardiovascular events, or to the final survey date among those without such events. For the NHANES dataset, CVD status was identified according to self-reported answers derived from the Medical Conditions Questionnaire (MCQ 160 series) [35]. Subjects were categorized as having prevalent CVD if they affirmed any of the corresponding items (MCQ160, MCQ160b, MCQ160c), which referred to physician-diagnosed coronary heart disease, congestive heart failure, angina, myocardial infarction, or stroke.

### 2.5. Covariates

Covariates were selected a priori based on established cardiovascular risk factors, consistent with previous studies of CMI and the sarcopenia index in relation to CKM syndrome [14,19]. Sociodemographic factors (age, sex, education, residence, marital status), lifestyle factors (smoking, drinking), and clinical/biochemical factors (diabetes, hypertension, LDL-C, BUN, UA, CRP) were adjusted for. Participants were classified as hypertensive if they exhibited high blood pressure (systolic blood pressure ≥ 130 mmHg or diastolic blood pressure ≥ 80 mmHg), self-reported hypertension diagnosed by a physician, or were taking antihypertensive drugs [36]. Diabetes was determined if any one of the following criteria was met: fasting glucose ≥ 126 mg/dL, glycated hemoglobin ≥ 6.5%, self-reported doctor-diagnosed diabetes, or current use of antidiabetic treatments [37]. In both the NHANES and CHARLS databases, all variables were gathered by trained research staff following standardized procedures, covering sociodemographic profiles, health-related information, and fasting venous blood specimens for biochemical assays. We evaluated collinearity between the adjusted variables by applying the variance inflation factor (VIF), and no substantial multicollinearity was detected (Tables S3–S5).

### 2.6. Statistical Analysis

#### 2.6.1. Descriptive Statistics and Missing Data

Participants were stratified according to baseline quartiles of CMI-MLI for subsequent analyses. Descriptive statistics for CHARLS and NHANES were analyzed separately using identical protocols, with baseline socio-demographic and clinical traits shown as mean ± SD for continuous variables, and as numbers with percentages (n (%)) for categorical variables. Considering the low proportion of incomplete covariates, multiple imputation was performed to account for missing data, rather than excluding individuals with incomplete observations, to maximize statistical power. Missing covariate values were imputed using multiple imputation by chained equations (MICE) with predictive mean matching (mice package, version 3.19.0, m = 5, seed = 1234) [38]. Results were pooled across the five imputed datasets using Rubin’s rules. Because the proportion of missing data was very low in the CHARLS cohort (≤0.02% for all covariates), the pooled estimates were virtually identical to those obtained from a single completed dataset. The percentage of missing data for each covariate in the CHARLS and NHANES databases is summarized in Tables S6 and S7.

#### 2.6.2. Cross-Sectional Analysis

CHARLS served as the primary unweighted analytical cohort, in which logistic regression was used to explore the cross-sectional link between CMI-MLI with advanced CKM syndrome (Stages 3 and 4). This cross-sectional association was further replicated in NHANES using weighted logistic regression. For NHANES, combined sample weights were computed as two-thirds WTSSCB4Y (1999–2002) and one-third WTSSCB2Y (2003–2004), and sampling weights, strata, and primary sampling units (PSUs) were incorporated into models to account for the intricate survey sampling framework.

#### 2.6.3. Longitudinal Analysis

For the prospective analysis of CVD incidence in the CHARLS cohort, event frequencies were expressed as incident cases per 1000 person-years. We employed the Kaplan–Meier method to estimate the cumulative likelihood of CVD, and between-stratum differences were tested by the log-rank test. Cox regression models were adopted to explore the association between CMI-MLI with the onset of CVD during the follow-up period. CMI-MLI was analyzed both as a continuous variable and as a categorical variable using a four-level ranking scheme. Three nested regression models were established: a crude model with no covariates; Model 1 adjusted for age, gender, residential region, educational attainment, and marital condition; and Model 2 further adjusted for alcohol consumption, smoking habit, uric acid (UA), blood urea nitrogen (BUN), C-reactive protein (CRP), diabetes mellitus (DM), hypertension, and low-density lipoprotein cholesterol (LDL-C). This sequential strategy was used to evaluate the influence of incremental covariate adjustment on the association. To formally assess whether CMI-MLI provided prognostic information beyond CKM stage, we compared nested models using a likelihood-ratio test. The prospective association of CMI-MLI with new-onset CVD was reported using hazard ratios (*HR*s) and their corresponding 95% confidence intervals (*CI*s). Restricted cubic spline (RCS) with three pre-specified knot points was conducted to detect potential nonlinear relationships; the knot number was chosen to balance model flexibility and reduce overfitting, in accordance with previous methodological reports [39]. To investigate interaction effects, subjects were divided into four subgroups according to median CMI and MLI values. The median was chosen as a natural, data-driven cut point that yields balanced group sizes, consistent with previous studies of joint associations in CKM syndrome [40]. To formally assess whether CMI and MLI interact multiplicatively, we additionally fitted Cox models with a CMI × MLI interaction term, both as continuous variables and as binary categories by median split. To justify the single-index (equal-weight) formulation of CMI-MLI, we compared the composite index against models in which ln (CMI) and ln (MLI) were entered as separate terms, as well as against models containing either component alone, using likelihood-ratio tests and the Akaike information criterion (AIC) and Bayesian information criterion (BIC).

#### 2.6.4. Predictive Performance

Receiver operating characteristic (ROC) curves were constructed, and DeLong’s method was used to assess the area under the curve (AUC) values of CMI, MLI, and the combined CMI-MLI index for predicting CVD. The predictive performance of CMI-MLI was also compared with several established cardiovascular risk markers, including the triglyceride glucose index (TyG), atherogenic index of plasma (AIP), and TyG-WHtR. The discriminatory performance was further assessed by bootstrap internal validation (200 resamples) using the rms package, version 8.1.1, and the incremental value of CMI-MLI over a model containing covariates and CKM stage was quantified by the integrated discrimination improvement (IDI) using the survIDINRI package, version 1.1.2.

#### 2.6.5. Subgroup and Sensitivity Analyses

In addition, stratified analyses were performed across subgroups defined by age (<60, ≥60 years), gender, residential area, marital status, drinking, smoking, hypertension, and diabetes mellitus. Likelihood ratio tests were employed to evaluate multiplicative interactions, and subgroup results were presented using forest plots. To assess the robustness of our findings, we performed the following sensitivity analyses: (i) inclusion of participants with extreme values (i.e., the 1st and 99th percentiles) to examine the influence of outliers; (ii) exclusion of individuals with any missing values; (iii) exclusion of the 2020 CHARLS survey cycle to avoid potential confounding from the COVID-19 pandemic; (iv) a Fine-Gray competing-risk model treating all-cause mortality as the competing event; (v) further adjustment for the frailty index (Fried physical frailty phenotype) and hand grip strength; and (vi) a landmark analysis excluding CVD events occurring within the first 2 years of follow-up to address potential reverse causation. All statistical analyses were performed using R (Version 4.5.0), and a two-tailed test was applied, with statistical significance defined as *p* < 0.05.

## 3. Results

### 3.1. Characteristics of Participants

After applying exclusion criteria, 6921 participants from the CHARLS dataset (46.87% male, mean age 60.76 ± 9.91 years) and 3185 individuals from the 1999–2004 NHANES dataset (46.00% male, mean age 60.00 ± 11.00 years) formed part of the baseline analysis. Table 1 presents the baseline profiles of subjects in the CHARLS cohort stratified by quartiles of CMI-MLI levels. In both cohorts, subjects classified into the highest CMI-MLI quartile were characterized by older age, female sex, marriage, rural residence, and elementary or lower educational attainment. Additionally, they had a lower tendency to consume alcohol and exhibited poorer metabolic profiles than those in the lowest quartile. Individuals with elevated CMI-MLI values showed a higher frequency of hypertension, DM, and dyslipidemia. In the CHARLS dataset, current smoking prevalence decreased across ascending CMI-MLI quartiles, whereas no such consistent association was observed in the NHANES dataset. Detailed information regarding the baseline features of CHARLS participants without prevalent CVD at baseline is presented in Table S8, and those of NHANES participants are presented in Table S9. Within the CHARLS cohort, the proportions of participants in CKM stages 0 to 4 were 6.08%, 13.84%, 31.56%, 34.47%, and 14.04%, respectively (Table 1). Notably, in comparison with individuals possessing lower CMI-MLI levels, participants with elevated values had a reduced prevalence of CKM stages 0–1 and an increased proportion of stages 2–4. Moreover, the prevalence of advanced CKM stages climbed gradually with ascending CMI-MLI quartiles, and a similar trend was also observed in the NHANES dataset (Figure S2).

### 3.2. Association Between CMI-MLI and Advanced CKM Stages at Baseline

**Figure 2:**
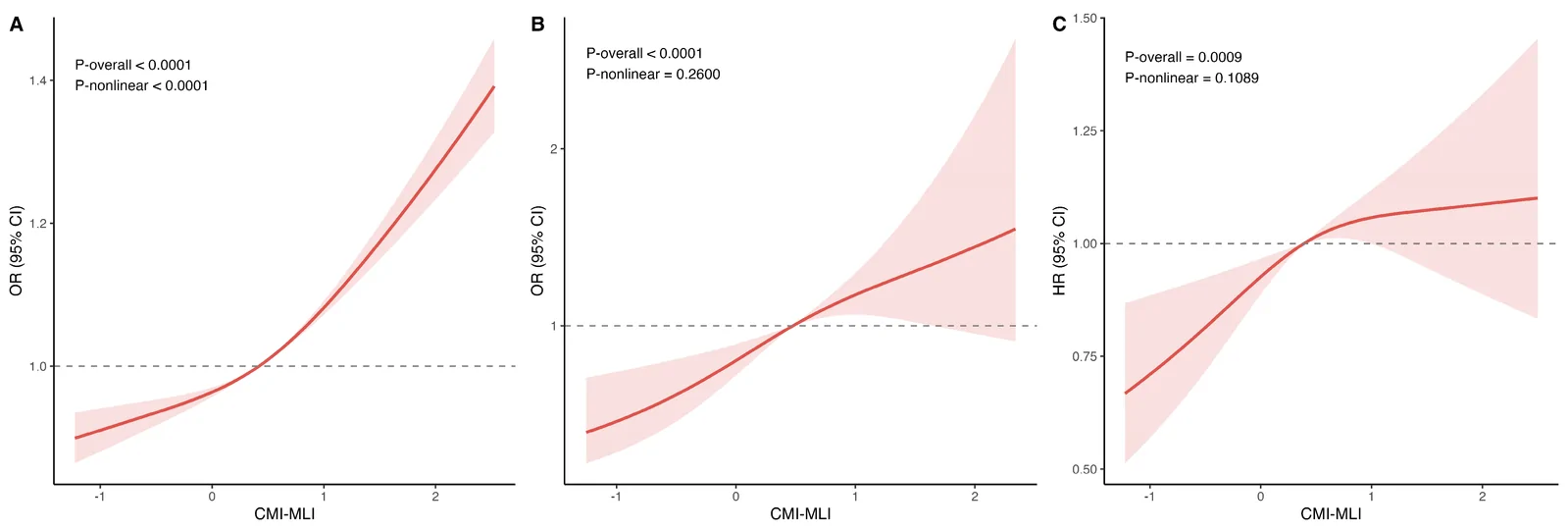
Restricted cubic spline (RCS) analysis after full adjustment. (**A**) Advanced cardiovascular-kidney-metabolic stages, CHARLS cohort; (**B**) Advanced cardiovascular-kidney-metabolic stages, NHANES cohort; (**C**) Incident cardiovascular disease, CHARLS cohort. RCS was fitted with three knots placed at the 10th, 50th, and 90th percentiles of CMI-MLI. Models were adjusted for age, sex, residence place, education, marital status, drinking, smoking, uric acid, blood urea nitrogen, C-reactive protein, diabetes, hypertension, and low-density lipoprotein cholesterol. Shaded areas represent 95% confidence intervals. Abbreviations: CMI-MLI, cardiometabolic index-muscle-loss index; CHARLS, China Health and Retirement Longitudinal Study; NHANES, National Health and Nutrition Examination Survey.

First, multivariable logistic regression analyses in the CHARLS (Table 2) cohort demonstrated that CMI-MLI was independently associated with more severe CKM stages after adjustment for all covariates. Accordingly, each 1-standard deviation (SD) increment in CMI-MLI corresponded to an odds ratio (OR) of 2.03 (95% CI 1.88–2.20) for advanced CKM stages. Compared with the lowest CMI-MLI quartile (Q1), notably increased risks of advanced CKM stages were found in Q2, Q3, and Q4, accompanied by a *p* for trend of < 0.0001. In a sensitivity analysis defining Stage 3 solely by G4/G5 CKD, the association was substantially attenuated (per-SD OR 1.086, 95% CI 1.008–1.171, *p* = 0.031; quartile analysis non-significant, with Q4 OR 1.198, 95% CI 0.971–1.478, *p* = 0.092), indicating that the cross-sectional association was largely driven by the overlap between the CMI components and the Framingham risk score (Table S10). Multivariable logistic regression analyses performed in the NHANES dataset replicated the cross-sectional association (per-SD OR 1.34, 95% CI 1.19–1.50; Table S11). In addition, multivariable-adjusted RCS analyses revealed a graded association between CMI-MLI and the risk of advanced CKM stages: a nonlinear positive correlation was identified in the CHARLS cohort (*p* for non-linearity < 0.0001, Figure 2A), while a monotonically increasing linear relation was confirmed in the NHANES dataset (*p* for non-linearity = 0.260, Figure 2B).

### 3.3. Association Between Baseline CMI-MLI and New-Onset CVD Among Participants with CKM Stages 0–3

In the CHARLS study, over a median follow-up of 9 years (interquartile range 7–9 years), 1384 new-onset CVD events were documented among 5768 participants over a total of 43,490 person-years of follow-up, corresponding to an unadjusted event incidence of 31.82 per 1000 person-years. As shown in the Kaplan–Meier analysis, the cumulative incidence of CVD increased progressively from the lowest to the highest CMI-MLI quartile. At 9 years, the cumulative incidence was 20.4% in Q1, 26.3% in Q2, 28.3% in Q3, and 30.0% in Q4. The four curves separated early and remained distinct throughout follow-up (log-rank *p* < 0.0001, Figure S3). Later, Cox regression analyses were utilized to investigate the link between baseline CMI-MLI with incident CVD risk. Table 3 shows the relationships between CMI-MLI and CVD incidence under three progressively adjusted Cox regression models. After full adjustment, each SD increase in CMI-MLI corresponded to an 11% higher risk of incident CVD (HR 1.11, 95% CI 1.04–1.17). Compared with the lowest quartile (Q1), participants in the higher quartiles showed incrementally elevated risks of incident CVD (*p* for trend = 0.001; Table 3). Furthermore, multivariable-adjusted RCS revealed a monotonically increasing linear relationship of CMI-MLI with CVD hazard (*p* for nonlinearity = 0.1089, Figure 2C). Adding CMI-MLI to a fully adjusted Cox model that already included CKM stage significantly improved model fit (likelihood ratio test, *p* = 0.019), indicating that CMI-MLI provides prognostic information beyond CKM stage. Formal model comparison supported the composite formulation. The constrained single-index model did not fit the data significantly worse than a two-term model containing ln (CMI) and ln (MLI) as separate terms (likelihood-ratio test, χ^2^ = 0.068, df = 1, *p* = 0.795), and it yielded the lowest AIC (23,181.78) and BIC (23,265.50) among models containing neither component, either component alone, or both components separately (Table S12). The composite significantly improved model fit relative to the covariates-only model (χ^2^ = 12.014, df = 1, *p* = 0.0005).

### 3.4. Joint Effects of CMI and MLI on the Risk of CVD in Individuals with CKM Stages 0–3

Within the CHARLS cohort, all subjects were categorized into four subgroups based on the median cut-offs of CMI and MLI (Table S13). After full adjustment in the Cox model, comparisons were conducted with the reference category (low CMI plus low MLI). Neither the high CMI plus low MLI group (HR = 1.16, 95% CI = 0.99–1.36, *p* = 0.0731) nor the low CMI plus high MLI group (HR = 1.05, 95% CI = 0.89–1.24, *p* = 0.5707) demonstrated a statistically significant correlation with incident CVD risk. Although a marginal increasing trend in CVD risk was observed in the high CMI plus low MLI group, the low CMI plus high MLI group exhibited no such trend. Notably, individuals exhibiting simultaneously elevated CMI and MLI values showed a significantly increased risk of developing CVD (HR = 1.23, 95% CI = 1.03–1.46, *p* = 0.0195). Formal interaction testing revealed no statistically significant multiplicative interaction between CMI and MLI, either as continuous variables (*p* = 0.510) or as binary categories by median split (*p* = 0.934; Table S14). On the additive scale, the relative excess risk due to interaction (RERI) was 0.019 (95% CI −0.215 to 0.253), the attributable proportion was 1.6%, and the synergy index was 1.09, likewise indicating no statistically significant additive interaction.

### 3.5. Predictive Performance of CMI-MLI on the Risk of CVD in Individuals with CKM Stages 0–3

**Figure 3:**
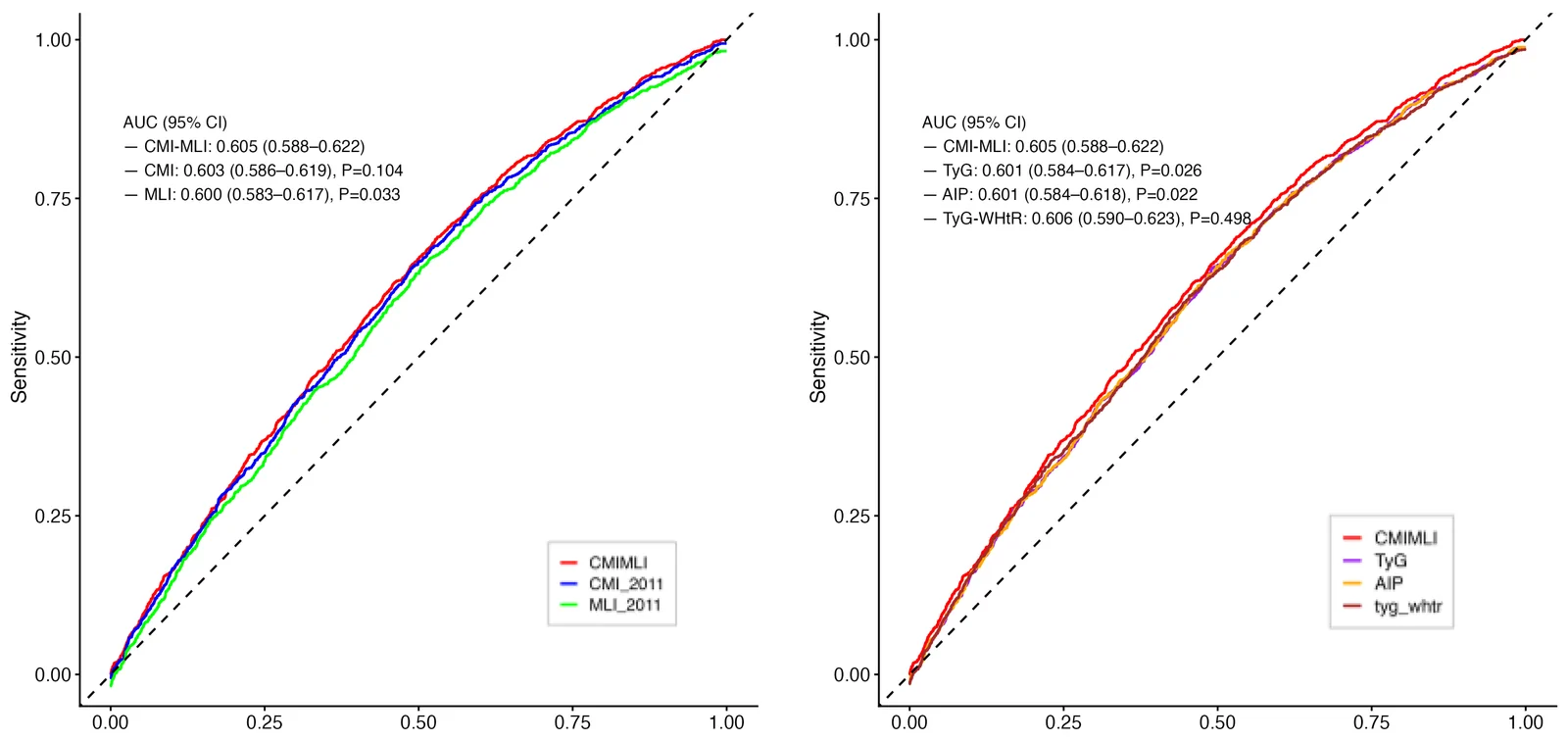
Receiver operating characteristic (ROC) curve analysis for the predictive ability of CMI-MLI and related indexes of cardiovascular disease risk among participants with cardiovascular-kidney-metabolic stages 0–3. (**A**) CMI-MLI, CMI, and MLI; (**B**) CMI-MLI, TyG, AIP, and TyG-WHtR. Area under the curve (AUC) values were: CMI-MLI 0.605 (95% CI 0.588–0.622); CMI 0.603 (95% CI 0.586–0.619; DeLong *p* = 0.104); MLI 0.600 (95% CI 0.583–0.617; DeLong *p* = 0.033); TyG 0.601 (95% CI 0.584–0.617; DeLong *p* = 0.026); AIP 0.601 (95% CI 0.584–0.618; DeLong *p* = 0.022); TyG-WHtR 0.606 (95% CI 0.590–0.623; DeLong *p* = 0.498). All adjusted for age, sex, residence place, education, marital status, drinking, smoking, uric acid, blood urea nitrogen, C-reactive protein, diabetes, hypertension, and low-density lipoprotein cholesterol. Abbreviations: CMI, cardiometabolic index; MLI, muscle-loss index; CMI-MLI, cardiometabolic index-muscle-loss index; TyG, Triglyceride-Glucose Index; AIP, Atherogenic Index of Plasma; TyG-WHtR, Triglyceride-Glucose-Waist-to-Height Ratio; AUC, area under the curve; ROC, receiver operating characteristic; CI, confidence interval.

ROC analyses were performed in the CHARLS cohort to compare the predictive ability of CMI, MLI, CMI-MLI, and several established markers (Figure 3). The AUCs with 95% confidence intervals and DeLong test results are presented in Table S15. CMI-MLI achieved an AUC of 0.605, which was comparable to that of CMI (0.603; DeLong *p* = 0.104) and TyG-WHtR (0.606; DeLong *p* = 0.498). The differences in AUC versus MLI (0.600; DeLong *p* = 0.033), TyG (0.601; DeLong *p* = 0.026), and AIP (0.601; DeLong *p* = 0.022) reached statistical significance, although the absolute differences were small (<0.01). Decision curve analysis showed that the net benefit of CMI-MLI was comparable to that of CMI and MLI alone across the range of threshold probabilities, with no clear incremental net benefit over CMI (Figure S4). Bootstrap internal validation yielded an optimism-corrected c-index of 0.604 (apparent 0.612), confirming that the discrimination of CMI-MLI was modest. The integrated discrimination improvement (IDI) of adding CMI-MLI to a model already containing covariates and CKM stage was 0.0013 (95% CI −0.0004 to 0.0034, *p* = 0.159), indicating no significant incremental discrimination.

### 3.6. Subgroup Analyses and Sensitivity Analyses

To further explore the association between CMI-MLI and incident CVD, we performed stratified and interaction assessments within the CHARLS cohort stratified by key potential confounding variables. As shown in Figure S5, we observed no significant interaction between the CMI-MLI index and all variables in the stratified subgroups. Similarly, all sensitivity analyses were also conducted within the CHARLS cohort to validate the stability of our primary outcomes. First, after including participants with extreme percentiles (1st and 99th) to examine outlier effects, results remained consistent with main findings (Table S16). Second, limiting analyses to subjects with complete covariate data to account for selection bias yielded similar results (Table S17). Third, omitting the 2020 wave to adjust for COVID-19-related disturbances produced largely unchanged findings (Table S18). In additional sensitivity analyses, the association of CMI-MLI with incident CVD remained consistent: the Fine-Gray subdistribution HR was 1.097 (95% CI 1.040–1.156, *p* = 0.00066); further adjustment for the frailty index and hand grip strength yielded an HR of 1.107 (95% CI 1.046–1.172, *p* = 0.00044); and the landmark analysis excluding events within the first 2 years yielded an HR of 1.088 (95% CI 1.018–1.163, *p* = 0.013) (Table S19). Furthermore, pooling the five imputed datasets using Rubin’s rules yielded estimates virtually identical to those from the single completed dataset (pooled per-SD HR 1.106, 95% CI 1.045–1.170 for incident CVD; pooled per-SD OR 2.03, 95% CI 1.88–2.20 for advanced CKM stages; Table S20).

## 4. Discussion

Based on two national cohort studies, elevated CMI-MLI values were independently associated with a higher risk of incident CVD over a median follow-up of 9 years among participants with CKM syndrome but no baseline CVD. At baseline, CMI-MLI was also associated with advanced CKM stages, although this cross-sectional association should be interpreted in light of the partial overlap between the CMI components and the Framingham risk score. Compared with evaluating CMI and MLI individually, the combined CMI-MLI index provides a single, parsimonious marker of cardiovascular risk. To our knowledge, this is the first study to examine the association between CMI-MLI and CKM syndrome progression.

Sarcopenic obesity develops gradually with age, typified by progressive reductions in muscle mass and strength, together with relative or absolute gains in adipose tissue [20,23]. The coexistence of muscle loss and excess adiposity induces IR [41,42]. IR related to sarcopenic obesity further triggers chronic inflammation and oxidative stress [43], causing vascular smooth muscle hyperplasia and vascular endothelial injury [21]. These processes link sarcopenic obesity not only to CVD but also to a broad spectrum of metabolic disorders. Given the rising worldwide prevalence of obesity, metabolic syndrome and CKD—all key metabolic risk markers for CKM—this correlation carries particular significance [3]. Accumulating evidence also demonstrates that sarcopenic obesity predicts worse health outcomes, including functional disability, cardiovascular disease, other chronic comorbidities, and all-cause mortality, compared with obesity alone [44,45,46,47]. Furthermore, emerging evidence suggests that sarcopenic obesity accelerates the progression of CKM to more advanced stages [5,48]. Nevertheless, low skeletal muscle mass, abdominal obesity, and insulin resistance are closely linked at both mechanistic and epidemiological levels. Currently available biomarkers fail to combine cardiometabolic risk profiles with muscle-related characteristics, leading to insufficient identification of individuals with concurrent high-risk traits. Against this background, we constructed a novel CMI-MLI index that combines central obesity, IR (reflected by CMI), and muscle status (reflected by MLI). Our results suggest that increased CMI-MLI levels may act as a possible risk factor for new-onset CVD in participants with stage 0–3 CKM.

As outlined in the AHA clinical guidance, the central aim of CKM syndrome management is to prevent adverse cardiovascular events and halt or reverse its progression [49]. Accordingly, we conducted cross-sectional and longitudinal assessments to examine the relationship of the CMI-MLI index with CKM syndrome progression. Of note, we did not adopt the standard clinical definition of sarcopenic obesity; instead, we utilized elevated CMI (reflecting IR and abdominal obesity) coupled with high MLI (indicating muscle impairment) as a surrogate marker for this high-risk phenotype. In a supplementary analysis, MLI was significantly negatively correlated with hand grip strength in the CHARLS cohort (Spearman rho = −0.367, *p* < 0.0001). Although this correlation supports its interpretation as a marker of muscle impairment, it is only of moderate strength, indicating that MLI captures muscle-related risk imperfectly rather than directly measuring muscle mass. Given that stage 3 CKM constitutes a high-risk phenotype for adverse cardiovascular events, and stage 4 CKM is linked to an extremely high risk of CVD recurrence and mortality, we merged these two stages into advanced CKM stages, consistent with previous research [4]. In line with our hypothesis, logistic regression identified CMI-MLI as an independent predictor of advanced CKM stages. Cox regression further demonstrated its independent association with incident CVD among participants with CKM stages 0–3. The nonlinear positive link between CMI-MLI and advanced CKM stages in the CHARLS cohort suggested a potential threshold effect, whereas the linear association in the cross-sectional NHANES cohort and the longitudinal CVD analysis likely reflected consistent, cumulative risk effects. These differing patterns may be explained by differences in sample size and statistical power (CHARLS n = 6921 vs. NHANES n = 3185), population characteristics (Chinese vs. multiethnic US adults), and the distribution of metabolic risk factors between the two cohorts. Inspection of the restricted cubic spline curves in both cohorts revealed a smooth increase in risk across the CMI-MLI range without a distinct threshold; we therefore did not identify a specific cutoff. Our joint analysis of the CHARLS longitudinal cohort showed that the concurrent elevation of CMI and MLI conferred a significantly increased hazard of incident CVD, whereas individual assessment of either marker alone showed no significant association. This result aligns with a recent CHARLS-based study, which found that neither elevated CMI nor sarcopenia alone was associated with CVD, whereas their combination significantly predicted CVD events [50]. Similarly, Liu et al. reported that IR (assessed by TG/HDL-C ratio) and sarcopenia jointly increased cardiovascular risk in older adults [51]. Collectively, these findings suggest a joint association between CMI and MLI with incident CVD, consistent with prior evidence that the concurrent presence of both factors identifies individuals at higher cardiovascular risk. A limitation of the joint and interaction analyses is that dichotomizing CMI and MLI at their medians reduces statistical power and may obscure dose–response relationships. The null interaction findings—on both the multiplicative and additive scales—should therefore be interpreted cautiously, as a modest interaction could be missed by the dichotomized analysis. This limitation applies to the secondary joint analysis rather than the primary continuous analyses, in which the per-SD and restricted-cubic-spline models demonstrated a graded, linear dose–response association between CMI-MLI and incident CVD risk. Furthermore, ROC analysis showed that the discriminatory ability of CMI-MLI was modest (AUC 0.605) and comparable to that of CMI alone (0.603); although the AUC differences versus MLI, TyG, and AIP reached statistical significance, the absolute differences were all below 0.01 and therefore of negligible clinical significance. Formal model comparison showed that the composite index fitted the data as well as a two-term model containing ln(CMI) and ln(MLI) separately (likelihood-ratio test, *p* = 0.795), and outperformed MLI alone, although its advantage over CMI alone was modest (ΔAIC = 0.52). CMI therefore carried the predominant predictive signal, with MLI contributing a smaller increment; the value of the composite lies chiefly in integrating both cardiometabolic and muscle-related information into a single, parsimonious index rather than in materially improving discrimination. These findings suggest that the combined assessment of IR, abdominal obesity, and muscle impairment—as captured by the CMI-MLI index—may help identify individuals at elevated cardiovascular risk among patients with stage 0–3 CKM.

The biological mechanisms underlying this association are not fully understood. Sarcopenic obesity promotes systemic chronic inflammation and oxidative stress, which impair vascular endothelial function, worsen insulin resistance, and accelerate atherogenesis, ultimately predisposing to ischemic heart disease, arrhythmia, and heart failure [10,20,21,22]; a sedentary lifestyle may further amplify this low-grade inflammation. Lifestyle interventions and pharmacologic approaches targeting dyslipidemia and obesity may attenuate this risk [22,52,53,54]. From a clinical perspective, participants in the highest CMI-MLI quartile (Q4, CMI-MLI > 0.927) had the highest risk of incident CVD (HR 1.35), suggesting that this cut point may serve as a preliminary reference for identifying high-risk individuals. However, a formal clinical threshold requires validation in independent cohorts. We also acknowledge that cystatin C is not routinely measured in many primary care or resource-limited settings, and that its assay carries additional cost relative to creatinine alone; this limits both the immediate applicability and the cost-effectiveness of CMI-MLI in population-level screening. Its utility should therefore be further evaluated in settings where cystatin C testing is available and where the incremental clinical benefit justifies the added cost. Together, these findings support CMI-MLI as a measurable marker that may inform cardiovascular risk prevention and control.

Several major strengths characterize the present study. First, we provide novel evidence regarding the association between baseline CMI-MLI levels and the hazard of CVD, specifically in participants diagnosed with CKM syndrome. To ensure the reliability of our conclusions, comprehensive covariate-controlled models and varied sensitivity evaluations were employed to confirm the stability of the detected relationships. Second, CMI-MLI is constructed using only routine laboratory parameters and simple anthropometric measurements that are readily available in daily clinical practice. This feature allows the index to be conveniently incorporated into standard clinical assessments without additional costs or major modifications to existing workflows. Taken together, these findings suggest that CMI-MLI, as a simple and inexpensive marker, may modestly complement existing risk assessment, although its clinical utility requires external validation. Although our findings provide new perspectives on the link between CMI-MLI and cardiovascular risk, several potential limitations should be acknowledged in the present study. First, given its observational design, causal conclusions cannot be established. Even after extensive adjustment for multiple confounders, residual and unmeasured confounding could not be fully excluded. Our results therefore represent statistical associations rather than causal relationships, highlighting the need for future randomized controlled trials to validate these observations. Second, constrained by data availability, CKD staging was determined exclusively by eGFR without inclusion of albuminuria, and direct imaging measures of subclinical CVD were not available. We therefore classified Stage 3 using risk equivalents (G4/G5 CKD or a Framingham-predicted 10-year risk ≥ 21.6% in men/≥21.5% in women). The absence of UACR and direct imaging for subclinical CVD is a major limitation of our cross-sectional findings: a high predicted risk is not equivalent to established subclinical CVD, and this may have misclassified some individuals as Stage 3 and attenuated the cross-sectional association between CMI-MLI and advanced CKM stages. Furthermore, because the Framingham risk score shares HDL-cholesterol with the CMI component, the cross-sectional association between CMI-MLI and advanced CKM stages may be partly circular. Consistent with this concern, Stage 3 was classified almost entirely by the Framingham high-risk criterion (2379 of 2386 participants). Because G4/G5 CKD was uncommon in this community-based population (only 7 of 2386 Stage 3 participants), a sensitivity analysis defining Stage 3 solely by G4/G5 CKD effectively reduced the advanced group to prevalent CVD (Stage 4, n = 972) plus these seven participants, and substantially attenuated the association (per-SD OR 1.086, 95% CI 1.008–1.171, versus 2.03 in the main analysis). This attenuation confirms that the stronger original association was largely driven by the risk-score-based classification of Stage 3. The remaining association reflects the link between CMI-MLI and physician-confirmed prevalent CVD, which is not affected by this circularity and remained statistically significant. This limitation does not affect the prospective analysis of incident CVD, which used physician-confirmed events. The Stage 3 definition is reported transparently in the Methods. Third, the MLI index (Cys C/Cr) is a surrogate biochemical indicator rather than a direct measure of skeletal muscle mass (e.g., by dual-energy X-ray absorptiometry) or a confirmed diagnostic criterion for sarcopenia, and its construct validity is only moderate (handgrip-strength correlation, ρ = −0.367). Both cystatin C and creatinine are influenced by renal function, age, inflammation, and nutritional status including protein intake; accordingly, MLI may partly reflect these factors in addition to muscle mass, and we interpreted it as a composite marker of muscle-related and metabolic risk rather than a direct measure of muscle mass loss. Of note, our fully adjusted models already included uric acid, blood urea nitrogen, and C-reactive protein, providing partial adjustment for renal function and inflammation. In addition, since cystatin C is not routinely measured in general care or resource-constrained settings, its clinical applicability in population-level screening warrants further validation before broad implementation. Furthermore, some components of the CMI (waist circumference, triglycerides, and glucose-related measures) overlap with the metabolic criteria used in CKM staging. This conceptual overlap may partly contribute to the cross-sectional association between CMI-MLI and advanced CKM stages. However, in the prospective analysis of incident CVD, adding CMI-MLI to a model that already included CKM stage significantly improved model fit (likelihood ratio test, *p* = 0.019), suggesting that CMI-MLI provides prognostic information beyond the CKM staging itself. Fourth, although we used a two-dataset strategy, the NHANES dataset was cross-sectional and therefore provided only cross-sectional replication of the association with advanced CKM stages, rather than prognostic validation. The prognostic value of CMI-MLI for new-onset CVD in participants with CKM stages 0–3 was supported solely by the prospective CHARLS cohort. Finally, although we adjusted for major recognized covariates, residual confounding from several unmeasured factors cannot be fully excluded, including physical activity (missing in 75% of participants), dietary patterns (not comprehensively collected in CHARLS), lipid-lowering medication use (missing in 96.5% of participants; LDL-cholesterol was adjusted as an objective lipid-related covariate), family history of cardiovascular disease and duration of diabetes or hypertension (not available in CHARLS), and socioeconomic status (income data missing in 93%; educational attainment was used as the primary indicator). Notably, lipid-lowering medications directly lower triglycerides and raise HDL-cholesterol—both components of CMI—so their incomplete capture could bias the CMI-MLI calculation itself. Because physical activity, dietary quality, and medication adherence are generally associated with a lower risk of CVD and may also be correlated with lower CMI-MLI, their incomplete capture could lead to either over- or underestimation of the observed associations. Nevertheless, the association remained robust after additional adjustment for the frailty index and hand-grip strength (surrogates of physical capacity) and in a landmark analysis excluding early CVD events, suggesting that residual confounding from these lifestyle and socioeconomic factors is unlikely to fully explain our findings. In addition, our analysis used baseline values and did not account for time-varying changes in medication use or lifestyle factors during the 9-year follow-up, which could bias the longitudinal results. Nevertheless, reverse causation is unlikely, as the association remained significant after excluding early CVD events in the landmark analysis. Additionally, our findings were derived from middle-aged and older adults in two specific cohorts (CHARLS in China and NHANES in the US), and may not be generalizable to younger populations or other ethnic groups. Validation in additional prospective cohorts is warranted. Future studies should (1) validate CMI-MLI in prospective cohorts with urinary albumin-to-creatinine ratio and direct measures of muscle mass (e.g., dual-energy X-ray absorptiometry or bioimpedance); (2) examine whether CMI-MLI improves risk reclassification beyond established cardiovascular risk scores; and (3) evaluate time-varying changes in CMI-MLI and their association with cardiovascular outcomes.

## 5. Conclusions

In summary, among middle-aged and older adults, elevated CMI-MLI values were an independent risk indicator for incident CVD over a median 9-year follow-up in participants with CKM stages 0 to 3. At baseline, CMI-MLI was also associated with advanced CKM stages, although this cross-sectional association should be interpreted in light of the partial overlap between the CMI components and the Framingham risk score. Although its discriminatory ability was modest and its incremental value over CMI alone was limited, CMI-MLI may serve as a simple, parsimonious marker that simultaneously captures cardiometabolic and muscle-related risk. Given the observational design, these findings should be interpreted cautiously, and further prospective and interventional studies are warranted to evaluate the clinical utility of CMI-MLI.

## Figures and Tables

**Table 1:** Baseline characteristics of participants stratified by cardiometabolic index-muscle-loss index quartiles.

Characteristics	Overall	Quartiles of CMI-MLI			
		Q1	Q2	Q3	Q4
n	6921	1730	1730	1728	1733
Age, years	60.76 ± 9.91	60.35 ± 10.06	60.78 ± 9.88	60.94 ± 9.97	60.98 ± 9.73
Sex, n (%)					
male	3244 (46.87)	1062 (61.39)	858 (49.60)	733 (42.42)	591 (34.10)
female	3677 (53.13)	668 (38.61)	872 (50.40)	995 (57.58)	1142 (65.90)
Marital status, n (%)					
non-married	947 (13.68)	212 (12.25)	224 (12.95)	257 (14.87)	254 (14.66)
married	5974 (86.32)	1518 (87.75)	1506 (87.05)	1471 (85.13)	1479 (85.34)
Education, n (%)					
elementary school or below	5007 (72.35)	1202 (69.48)	1283 (74.16)	1260 (72.92)	1262 (72.82)
secondary school	1815 (26.22)	495 (28.61)	423 (24.45)	447 (25.87)	450 (25.97)
college and above	99 (1.43)	33 (1.91)	24 (1.39)	21 (1.22)	21 (1.21)
Residence place, n (%)					
rural	4498 (64.99)	1194 (69.02)	1177 (68.03)	1095 (63.37)	1032 (59.55)
urban	2423 (35.01)	536 (30.98)	553 (31.97)	633 (36.63)	701 (40.45)
Smoking, n (%)					
never	4183 (60.44)	871 (50.35)	1005 (58.09)	1127 (65.22)	1180 (68.09)
previous	656 (9.48)	191 (11.04)	166 (9.60)	150 (8.68)	149 (8.60)
current	2082 (30.08)	668 (38.61)	559 (32.31)	451 (26.10)	404 (23.31)
Drinking, n (%)					
no	4717 (68.15)	986 (56.99)	1153 (66.65)	1259 (72.86)	1319 (76.11)
yes	2204 (31.85)	744 (43.01)	577 (33.35)	469 (27.14)	414 (23.89)
BMI, kg/m^2^	23.49 ± 3.92	21.72 ± 3.43	22.71 ± 3.55	24.11 ± 3.80	25.42 ± 3.85
WC, cm	84.60 ± 12.27	77.13 ± 13.50	82.82 ± 10.38	87.26 ± 10.09	91.19 ± 10.04
SBP, mmHg	131.97 ± 21.99	127.67 ± 21.53	130.17 ± 20.77	133.80 ± 22.63	136.23 ± 22.02
DBP, mmHg	75.85 ± 12.14	73.71 ± 12.09	74.97 ± 11.91	76.83 ± 12.20	77.88 ± 11.95
FPG, mg/dL	110.30 ± 36.42	102.83 ± 25.14	106.48 ± 31.73	111.70 ± 40.60	120.18 ± 43.01
HbA1c, %	5.28 ± 0.86	5.16 ± 0.73	5.22 ± 0.77	5.31 ± 0.89	5.43 ± 0.99
TC, mg/dL	194.28 ± 38.57	189.33 ± 35.46	188.97 ± 36.18	198.03 ± 39.34	200.79 ± 41.61
TG, mg/dL	128.31 ± 80.70	67.86 ± 22.62	94.30 ± 26.77	129.51 ± 38.02	221.41 ± 99.37
HDL-C, mg/dL	51.24 ± 14.71	64.95 ± 14.23	54.43 ± 11.00	47.32 ± 9.48	38.27 ± 8.36
LDL-C, mg/dL	118.07 ± 34.77	112.96 ± 30.85	117.57 ± 31.92	126.21 ± 35.87	115.57 ± 38.50
CRP, mg/dL	2.68 ± 6.59	2.45 ± 6.77	2.33 ± 6.65	2.84 ± 6.83	3.08 ± 6.07
Cr, mg/dL	0.79 ± 0.25	0.83 ± 0.37	0.78 ± 0.18	0.78 ± 0.21	0.77 ± 0.21
CysC, mg/dL	1.03 ± 0.28	1.02 ± 0.32	1.03 ± 0.24	1.04 ± 0.28	1.02 ± 0.28
BUN, mg/dL	15.84 ± 4.64	16.63 ± 5.16	15.90 ± 4.39	15.53 ± 4.51	15.32 ± 4.34
UA, mg/dL	4.48 ± 1.27	4.41 ± 1.18	4.36 ± 1.25	4.46 ± 1.31	4.69 ± 1.31
eGFR, mL/min/1.73 m^2^	94.34 ± 14.86	94.48 ± 14.83	94.91 ± 13.94	94.30 ± 14.97	93.65 ± 15.63
CMI	1.64 ± 1.59	0.51 ± 0.16	0.92 ± 0.25	1.54 ± 0.47	3.58 ± 2.05
MLI	1.34 ± 0.32	1.27 ± 0.26	1.34 ± 0.29	1.37 ± 0.33	1.37 ± 0.39
CMI-MLI	0.44 ± 0.76	−0.51 ± 0.29	0.16 ± 0.15	0.68 ± 0.15	1.43 ± 0.39
MetS, n (%)	1955 (28.25)	54 (3.12)	153 (8.84)	473 (27.37)	1275 (73.57)
Hypertension, n (%)	2986 (43.14)	552 (31.91)	660 (38.15)	826 (47.80)	948 (54.70)
Dyslipidemia, n (%)	2795 (40.38)	263 (15.20)	381 (22.02)	748 (43.29)	1403 (80.96)
DM, n (%)	1056 (15.26)	161 (9.31)	189 (10.92)	292 (16.90)	414 (23.89)
CKM syndrome stages, n (%)					
Stage 0	421 (6.08)	264 (15.26)	127 (7.34)	29 (1.68)	1 (0.06)
Stage 1	958 (13.84)	419 (24.22)	346 (20.00)	171 (9.90)	22 (1.27)
Stage 2	2184 (31.56)	405 (23.41)	534 (30.87)	647 (37.44)	598 (34.51)
Stage 3	2386 (34.47)	447 (25.84)	507 (29.31)	638 (36.92)	794 (45.82)
Stage 4	972 (14.04)	195 (11.27)	216 (12.49)	243 (14.06)	318 (18.35)
Advanced CKM stages, n (%)	3358 (48.52)	642 (37.11)	723 (41.79)	881 (50.98)	1112 (64.17)

Data were expressed as mean ± SD or numbers (percentages). *p* values were derived from the Kruskal–Wallis test for continuous variables and the chi-square test for categorical variables. Participants were categorized into four groups according to the quartiles of baseline cardiometabolic index-muscle-loss index (Q1 ≤ −0.134, −0.134 < Q2 ≤ 0.391, 0.391 < Q3 ≤ 0.927, Q4 > 0.927). Abbreviations: BMI, body mass index; WC, waist circumference; SBP, systolic blood pressure; DBP, diastolic blood pressure; FPG, fasting plasma glucose; HbA1c, glycated hemoglobin; TC, total cholesterol; TG, triglyceride; HDL-C, high-density lipoprotein cholesterol; LDL-C, low-density lipoprotein cholesterol; CRP, C-reactive protein; Cr, creatinine; CysC: Cystatin C; BUN: Blood Urea Nitrogen; UA, Uric Acid; eGFR: estimated Glomerular Filtration Rate; CMI, cardiometabolic index; MLI, muscle-loss index; CMI-MLI, cardiometabolic index-muscle-loss index; DM, diabetes; MetS, metabolic syndrome; CKM, cardiovascular-kidney-metabolic.

**Table 2:** Association between cardiometabolic index-muscle-loss index and advanced cardiovascular-kidney-metabolic stages at baseline: CHARLS.

	Crude Model		Model 1		Model 2	
	OR (95% CI)	*p*	OR (95% CI)	*p*	OR (95% CI)	*p*
CMI-MLI per SD	1.56 (1.48, 1.64)	<0.0001	2.02 (1.89, 2.15)	<0.0001	2.03 (1.88, 2.20)	<0.0001
CMI-MLI quartile						
Q1	ref		ref		ref	
Q2	1.22 (1.06, 1.39)	0.005	1.47 (1.24, 1.73)	<0.0001	1.35 (1.11, 1.64)	0.002
Q3	1.76 (1.54, 2.02)	<0.0001	2.67 (2.25, 3.16)	<0.0001	2.13 (1.74, 2.61)	<0.0001
Q4	3.03 (2.64, 3.49)	<0.0001	6.11 (5.13, 7.29)	<0.0001	6.11 (4.93, 7.57)	<0.0001
*p* for trend		<0.0001		<0.0001		<0.0001

Crude model: unadjusted; Model 1: adjusted for age, sex, residence place, education and marital status; Model 2: adjusted for age, sex, residence place, education, marital status, drinking, smoking, uric acid, blood urea nitrogen, C-reactive protein, diabetes, hypertension and low-density lipoprotein cholesterol. *p* values were derived from the Wald test. Abbreviations: CMI-MLI, cardiometabolic index-muscle-loss index; SD, standard deviation; OR, odds ratio; CI, confidence interval; CHARLS: China Health and Retirement Longitudinal Study.

**Table 3:** Association between cardiometabolic index-muscle-loss index and the risk of incident cardiovascular disease in participants with cardiovascular-kidney-metabolic Stages 0–3: CHARLS.

	Crude Model		Model 1		Model 2	
	HR (95% CI)	*p*	HR (95% CI)	*p*	HR (95% CI)	*p*
CMI-MLI per SD	1.17 (1.11, 1.23)	<0.0001	1.15 (1.09, 1.21)	<0.0001	1.11 (1.04, 1.17)	<0.001
CMI-MLI quartile						
Q1	ref		ref		ref	
Q2	1.35 (1.15, 1.58)	<0.001	1.32 (1.12, 1.54)	<0.001	1.26 (1.07, 1.48)	0.01
Q3	1.44 (1.23, 1.68)	<0.0001	1.39 (1.18, 1.63)	<0.0001	1.25 (1.07, 1.47)	0.01
Q4	1.58 (1.36, 1.85)	<0.0001	1.51 (1.29, 1.77)	<0.0001	1.35 (1.15, 1.59)	<0.001
*p* for trend		<0.0001		<0.0001		0.001

Crude model: unadjusted; Model 1: adjusted for age, sex, residence place, education, marital status; Model 2: adjusted for age, sex, residence place, education, marital status, drinking, smoking, uric acid, blood urea nitrogen, C-reactive protein, diabetes, hypertension and low-density lipoprotein cholesterol. *p* values were derived from the Wald test. Abbreviations: CMI-MLI, cardiometabolic index-muscle-loss index; SD, standard deviation; HR, hazard ratio; CI, confidence interval.

## Data Availability

The datasets supporting the conclusions of this article are available in the China Health and Retirement Longitudinal Study (CHARLS) repository, https://charls.pku.edu.cn (accessed on 10 May 2026), and the U.S. National Health and Nutrition Examination Survey (NHANES) repository, https://www.cdc.gov/nchs/nhanes/index.html (accessed on 10 May 2026).

## Supplementary Materials

The following supporting information can be downloaded at: https://www.mdpi.com/article/10.3390/healthcare14193228/s1, Figure S1. Distribution of CMI-MLI in the study cohort. (A) Distribution in 5768 participants from the longitudinal CHARLS cohort; (B) Distribution in 6921 participants from the cross-sectional CHARLS cohort; (C) Distribution in 3185 participants from the cross-sectional NHANES cohort. Figure S2. Prevalence of cardiovascular-kidney-metabolic stages (A, C) and advanced cardiovascular-kidney-metabolic stages (B, D) by cardiometabolic index-muscle-loss index quartiles. (A) and (B) represent the China Health and Retirement Longitudinal Study (CHARLS) cohort, while (C) and (D) represent the National Health and Nutrition Examination Survey (NHANES) cohort. Figure S3. Kaplan–Meier curves for incident cardiovascular disease by cardiometabolic index-muscle-loss index quartiles among participants with cardiovascular-kidney-metabolic stages 0–3. Figure S4. Decision curve analysis for the net benefit of CMI-MLI, CMI, and MLI in predicting incident cardiovascular disease. Figure S5. Subgroup and interaction analyses for the association between CMI-MLI and cardiovascular disease among participants with cardiovascular-kidney-metabolic stages 0–3. CHARLS cohort, adjusted for age, sex, residence place, education, marital status, drinking, smoking, uric acid, blood urea nitrogen, C-reactive protein, diabetes, hypertension, and low-density lipoprotein cholesterol. Table S1. Unit conversion factors used in the calculation of CMI, MLI, and CMI–MLI. Table S2. Definition of CKM syndrome. Table S3. The results of the collinearity screening: CHARLS 2011. Table S4. The results of the collinearity screening: CHARLS 2011–2020. Table S5. The results of the collinearity screening: NHANES 1999–2004. Table S6. Pattern of missingness across model covariates: CHARLS. Table S7. Pattern of missingness across model covariates: NHANES. Table S8. Baseline characteristics of participants without baseline CVD stratified by cardiometabolic index-muscle-loss index quartiles. Table S9. Baseline characteristics of participants stratified by cardiometabolic index-muscle-loss index quartiles. NHANES 1999–2004. Table S10. Sensitivity analysis: association between CMI-MLI and advanced CKM stages defined solely by G4/G5 CKD (CHARLS). Table S11. Association between cardiometabolic index-muscle-loss index and advanced cardiovascular-kidney-metabolic stages at baseline: NHANES 1999–2004. Table S12. Model comparison for incident cardiovascular disease in the CHARLS cohort. Table S13. Joint associations of CMI and MLI with the risk of incident cardiovascular disease in participants with cardiovascular-kidney-metabolic Stages 0–3. Table S14. Multiplicative interaction between CMI and MLI on incident cardiovascular disease. Table S15. Predictive performance of CMI-MLI and other indices for incident cardiovascular disease in the CHARLS cohort. Table S16. Association of CMI-MLI with incident CVD within cardiovascular-kidney-metabolic stages 0–3, including participants with outlier values: CHARLS cohort. Table S17. Association of CMI-MLI with incident CVD within cardiovascular-kidney-metabolic stages 0–3, excluding participants with missing covariate data: CHARLS cohort. Table S18. Association of CMI-MLI with incident CVD within cardiovascular-kidney-metabolic stages 0–3, excluding 2020 wave participants: CHARLS 2011–2018. Table S19. Sensitivity analyses for the association between CMI-MLI and incident cardiovascular disease in the CHARLS cohort. Table S20. Comparison of single-dataset and pooled (Rubin’s rules) estimates in the CHARLS cohort.

## Abbreviations

AIP	Atherogenic index of plasma
AHA	American Heart Association
ASCVD	Atherosclerotic cardiovascular disease
AUC	Area under the curve
BMI	Body mass index
BUN	Blood urea nitrogen
CHARLS	China Health and Retirement Longitudinal Study
CI	Confidence interval
CMI-MLI	Cardiometabolic index-muscle-loss index
Cr	Creatinine
CRP	C-reactive protein
CVD	Cardiovascular disease
Cys C	Cystatin C
CKD	Chronic kidney disease
CKM	Cardiovascular-kidney-metabolic syndrome
DM	Diabetes mellitus
HDL-C	High-density lipoprotein cholesterol
HR	Hazard ratio
IR	Insulin resistance
LDL-C	Low-density lipoprotein cholesterol
NHANES	National Health and Nutrition Examination Survey
OR	Odds ratio
PIR	Poverty income ratio
PSUs	Primary sampling units
RCS	Restricted cubic spline
ROC	Receiver operating characteristic
SD	Standard deviation
SI	Sarcopenia index
TC	Total cholesterol
TG	Triglyceride
TyG	Triglyceride-glucose index
UA	Uric acid
VIF	Variance inflation factor
WHtR	Waist-to-height ratio
WC	Waist circumference
