# Supplementary material for: Association of Combined Cardiometabolic and Muscle Impairment Index with Cardiovascular-Kidney-Metabolic Syndrome Progression Among Middle-Aged and Older Adults: A Prospective Two-Cohort Study
Source: Healthcare (Basel). 2026 Oct 1;14(19):3228. doi: 10.3390/healthcare14193228 (PMC13665002; doi:10.3390/healthcare14193228)
Supplement: Supplementary file 1 [file healthcare-14-03228-s001.zip › Supplementary tables.pdf]

Table S1. Unit conversion factors used in the calculation of CMI, MLI, and CMI–MLI

**Measurement**	**SI Unit**	**Unit Used in Formula**	**Conversion Factor**	**Notes**
Triglycerides (TG)	mmol/L	mg/dL	× 88.57	Used in CMI calculation
High-Density Lipoprotein(HDL)	mmol/L	mg/dL	×38.67	Used in CMI calculation
height	cm	cm	–	Used in CMI calculation
waist	cm	cm	–	Used in CMI calculation
Creatinine (Cr)	μmol/L	mg/dL	÷ 88.4	Used in MLI calculation
Cystatin C (CysC)	mg/L	mg/L	–	Same unit as formula

Abbreviations:CMI, cardiometabolic index; MLI, muscle-loss index; CMI-MLI, cardiometabolic index-muscle-loss index;

Table S2. Definition of CKM syndrome

CKM syndrome stages	Definition
Stage 0: No CKM health risk factors	Participants with normal body mass index (BMI) (<23 kg/m2 for individuals with Asian ethnicity and <25 kg/m2 for other racial and ethnic groups), normal waist circumference (<80 and <90 cm for women and men with Asian race, respectively, and <88 and <102 cm for women and men in all other race and ethnicity categories, respectively) who did not meet criteria for the other stages.
Stage 1: Excess or dysfunctional adiposity	Individuals with overweight/obesity, abdominal obesity, or adipose tissue dysfunction without other metabolic risk factors, CKD, or subclinical/clinical CVD
	(1)BMI (≥23 kg/m^2 for individuals with Asian race and >25 kg/m^2 for all other race and ethnic groups)

	(2)WC ≥ 102 cm for men or ≥ 88 cm for women
	(3)FBG levels ranging from 100 to 124 mg/dL, or HbA1c levels between 5.7 and 6.4 %
Stage 2: Metabolic risk factors and moderate to high-risk CKD	Qualifying metabolic risk factors included elevated fasting serum triglycerides (≥135 mg/dL), hypertension, diabetes, or metabolic syndrome (≥3 of the following: elevated waist circumference, low high density lipoprotein cholesterol (HDL) level [<40 mg/dL or <50 mg/dL for men or women, respectively], fasting serum triglycerides ≥150 mg/dL, elevated blood pressure [systolic blood pressure ≥130, diastolic blood pressure ≥80 mmHg, and/or use of blood pressure-lowering medications], or prediabetes). CKD stages were identified based on GFR and urinary albumin-tocreatinine ratio.
Stage 3: Subclinical CVD in CKM	Presence of very-high-risk KDIGO CKD stages or a high-estimated 10-year cardiovascular disease (CVD) risk. The 10-year CVD risk was assessed using the AHA PREVENT equations for predicting cardiovascular events. A 10-year CVD risk of 20% or greater was classified as high risk.
Stage 4: Clinical CVD in CKM	Reported history of established cardiovascular conditions, including coronary heart disease, angina, myocardial infarction, heart failure, and cerebrovascular accident.

Abbreviations: CKM syndrome, Cardiovascular-Kidney-Metabolic syndrome; MeTS, metabolic syndrome; CKD, chronic kidney disease; CVD, cardiovascular disease; BMI, body mass index; WC, waist circumference; FBG, fasting blood glucose; HbA1c, glycated hemoglobin A1c; KDIGO, Kidney Disease Improving Global Outcomes; UACR, urine albumin-to-creatinine ratio; eGFR, estimated glomerular filtration rate; TG, triglycerides; SBP, systolic blood pressure; DBP, diastolic blood pressure.

Table S3. The results of the collinearity screening: CHARLS 2011

Variable	VIF
Age	8.976876121
Sex	2.173679459
Residence place	1.280843637
Education	1.142234036
Marital status	2.888407113

Drinking	1.428037729
Smoking	1.410769097
BUN	3.752343051
UA	4.213153819
CRP	1.086345081
DM	1.132256977
Hypertension	1.379296766
LDL-C	3.823015817

Abbreviations: CRP, C-reactive protein; UA, Uric Acid; BUN: Blood Urea Nitrogen; LDL-C, low-density lipoprotein cholesterol; DM, diabetes;

Table S4. The results of the collinearity screening: CHARLS 2011-2020

Variable	VIF
Age	1.157898864
Sex	1.553350156
Residence place	1.034268211
Education	1.057472927
Marital status	1.066034935
Drinking	1.138485759
Smoking	1.187490636
BUN	1.050100144
UA	1.136114552
CRP	1.009761353
DM	1.020512091
Hypertension	1.050618315
LDL-C	1.019682929

Abbreviations: CRP, C-reactive protein; UA, Uric Acid; BUN: Blood Urea Nitrogen; LDL-C, low-density lipoprotein cholesterol; DM, diabetes;

Table S5. The results of the collinearity screening: NHANES 1999-2004

Variable	VIF
Age	2.657430813
Sex	1.725638979
Family PIR	1.696680953
Education	1.936228876
Marital status	1.664910941
Race	1.6565506

Drinking	1.344705158
Smoking	1.773923936
BUN	1.54033521
UA	1.632160476
CRP	1.765325098
DM	1.548914131
Hypertension	1.872106641
LDL-C	1.904748274

Abbreviations: Family PIR, Family Poverty-to-Income Ratio; CRP, C-reactive protein; UA, Uric Acid; BUN: Blood Urea Nitrogen; LDL-C, low-density lipoprotein cholesterol; DM, diabetes;

Table S6. Pattern of missingness across model covariates: CHARLS

Covariate	Missing (*n*)	Missing (%)
Age	0	0.00
Sex	0	0.00
Marital status	0	0.00
Education	1	0.02
Residence place	0	0.00
Smoke	1	0.02
Drink	0	0.00
CRP	0	0.00
BUN	0	0.00
UA	0	0.00
LDL-C	0	0.00
Hypertension	0	0.00
DM	0	0.00

Abbreviations: CRP, C-reactive protein; UA, Uric Acid; BUN: Blood Urea Nitrogen; LDL-C, low-density lipoprotein cholesterol; DM, diabetes;

Table S7. Pattern of missingness across model covariates: NHANES

Covariate	Missing(*n*)	Missing (%)
Age	0	0.00
Sex	0	0.00
Marital status	102	3.20
Education	6	0.19
Race	0	0.00
Family PIR	278	8.73

Smoke	4	0.13
Drink	1166	36.61
CRP	1	0.03
BUN	0	0.00
UA	0	0.00
LDL-C	248	7.79
Hypertension	2	0.06
DM	2	0.06

Abbreviations: Family PIR, Family Poverty-to-Income Ratio; CRP, C-reactive protein; UA, Uric Acid; BUN: Blood Urea Nitrogen; LDL-C, low-density lipoprotein cholesterol; DM, diabetes;

Table S8. Baseline characteristics of participants without baseline CVD stratified by cardiometabolic index-muscle-loss index quartiles.

		Quartiles of CMI-MLI			
**Characteristics**	**Overall**	**Q1**	**Q2**	**Q3**	**Q4**
**n**	5768	1439	1446	1443	1440
**Age, years**	60.05 ± 9.78	59.48 ± 9.81	60.18 ± 9.84	60.14 ± 9.76	60.41 ± 9.68
**Sex, n(%)**					
male	2735(47.42)	881(61.22)	728(50.35)	624(43.24)	502(34.86)
female	3033(52.58)	558(38.78)	718(49.65)	819(56.76)	938(65.14)
**Marital status, n(%)**					
non-married	734(12.73)	168(11.67)	175(12.10)	191(13.24)	200(13.89)
married	5034(87.27)	1271(88.33)	1271(87.90)	1252(86.76)	1240(86.11)
**Education, n(%)**					
elementary school or below	4149(71.93)	986(68.52)	1064(73.58)	1048(72.63)	1051(72.99)
secondary school	1543(26.75)	428(29.74)	362(25.03)	378(26.20)	375(26.04)
college and above	76(1.32)	25(1.74)	20(1.38)	17(1.18)	14(0.97)
**Residence place, n(%)**					
rural	3839(66.56)	1008(70.05)	993(68.67)	941(65.21)	897(62.29)
urban	1929(33.44)	431(29.95)	453(31.33)	502(34.79)	543(37.71)
**Smoking, n(%)**					
Never	3492(60.54)	725(50.38)	832(57.54)	953(66.04)	982(68.19)
Previous	490(8.50)	138(9.59)	132(9.13)	113(7.83)	107(7.43)
Current	1786(30.96)	576(40.03)	482(33.33)	377(26.13)	351(24.38)
**Drinking, n(%)**					
no	3842(66.61)	794(55.18)	942(65.15)	1032(71.52)	1074(74.58)
yes	1926(33.39)	645(44.82)	504(34.85)	411(28.48)	366(25.42)
**BMI, kg/m^2**	23.38 ± 3.79	21.64 ± 3.20	22.70 ± 3.52	23.99 ± 3.60	25.18 ± 3.83

**WC,cm**	84.24 ± 12.01	77.01 ± 13.16	82.68 ± 10.31	86.84 ± 9.89	90.44 ± 10.00
**SBP, mmHg**	131.01 ± 21.57	126.71 ± 21.29	129.29 ± 20.18	132.36 ± 21.76	135.68 ± 21.99
**DBP, mmHg**	75.61 ± 12.00	73.59 ± 12.00	74.81 ± 11.79	76.43 ± 12.02	77.62 ± 11.78
**FPG, mg/dL**	109.56 ± 34.22	103.26 ± 25.28	106.39 ± 33.23	109.53 ± 31.92	119.08 ± 42.25
**HbA1c, %**	5.26 ± 0.83	5.15 ± 0.68	5.20 ± 0.75	5.30 ± 0.90	5.38 ± 0.94
**TC, mg/dL**	194.22 ± 38.38	189.74 ± 35.27	188.81 ± 36.57	198.02 ± 38.89	200.34 ± 41.24
**TG, mg/dL**	126.94 ± 80.23	67.09 ± 21.26	92.76 ± 25.59	128.50 ± 37.24	219.53 ± 99.80
**HDL-C, mg/dL**	51.53 ± 14.64	65.21 ± 14.27	54.55 ± 10.88	47.77 ± 9.36	38.59 ± 8.45
**LDL-C, mg/dL**	117.93 ± 34.65	113.21 ± 30.86	117.43 ± 32.19	125.90 ± 35.72	115.15 ± 38.03
**CRP, mg/dL**	2.50 ± 6.04	2.33 ± 6.66	2.32 ± 6.86	2.61 ± 5.68	2.74 ± 4.69
**Cr, mg/dL**	0.78 ± 0.24	0.82 ± 0.34	0.78 ± 0.18	0.77 ± 0.20	0.76 ± 0.19
**CysC, mg/dL**	1.01 ± 0.27	1.01 ± 0.30	1.02 ± 0.24	1.03 ± 0.27	1.00 ± 0.28
**BUN, mg/dL**	15.79 ± 4.52	16.52 ± 4.83	15.88 ± 4.44	15.51 ± 4.44	15.25 ± 4.25
**UA, mg/dL**	4.46 ± 1.25	4.39 ± 1.17	4.34 ± 1.22	4.44 ± 1.30	4.66 ± 1.28
**eGFR, mL/min/1.73m^2**	95.29 ± 14.36	95.36 ± 14.26	95.63 ± 13.60	95.39 ± 14.48	94.80 ± 15.05
**CMI**	1.60 ± 1.55	0.50 ± 0.16	0.90 ± 0.24	1.51 ± 0.45	3.49 ± 2.02
**MLI**	1.33 ± 0.32	1.26 ± 0.26	1.34 ± 0.28	1.37 ± 0.33	1.37 ± 0.40
**CMI-MLI**	0.41 ± 0.75	-0.52 ± 0.29	0.13 ± 0.15	0.65 ± 0.16	1.40 ± 0.39
**MetS, n(%)**	1472(25.52)	37(2.57)	115(7.95)	340(23.56)	980(68.06)
**Hypertension, n(%)**	2286(39.63)	411(28.56)	515(35.62)	628(43.52)	732(50.83)
**Dyslipidemia, n(%)**	2225(38.57)	206(14.32)	312(21.58)	574(39.78)	1133(78.68)
**DM, n(%)**	803(13.92)	125(8.69)	153(10.58)	212(14.69)	313(21.74)
**CKM syndrome stages, n (%)**					
Stage 0	412(7.14)	256(17.79)	122(8.44)	33(2.29)	1(0.07)
Stage 1	940(16.30)	395(27.45)	343(23.72)	178(12.34)	24(1.67)
Stage 2	2144(37.17)	378(26.27)	507(35.06)	642(44.49)	617(42.85)
Stage 3	2272(39.39)	410(28.49)	474(32.78)	590(40.89)	798(55.42)

Data were expressed as mean ± SD or numbers (percentages). Participants were categorized into four groups according to the quartiles of baseline cardiometabolic index-muscle-loss index. (Q1 ≤ -0.134, -0.134< Q2 ≤0.391, 0.391< Q3 ≤ 0.927 , Q4 > 0.927). P values were derived from the Kruskal-Wallis test for continuous variables and the chi-square test for categorical variables.

Abbreviations: BMI, body mass index; WC, waist circumference; SBP, systolic blood pressure; DBP, diastolic blood pressure; FPG, fasting plasma glucose; HbA1c, glycated hemoglobin; TC, total cholesterol; TG, triglyceride; HDL-C, high-density lipoprotein cholesterol; LDL-C, low-density lipoprotein cholesterol; CRP, C-reactive protein; Cr, creatinine; CysC: Cystatin C; BUN: Blood Urea Nitrogen; UA, Uric Acid; eGFR: estimated Glomerular Filtration Rate; CMI, cardiometabolic index; MLI, muscle-loss index; CMI-MLI, cardiometabolic index-muscle-loss index; DM, diabetes; MetS, metabolic syndrome; CVD, cardiovascular disease; CKM,

cardiovascular-kidney-metabolic.

Supplementary Table S9. Baseline characteristics of participants stratified by cardiometabolic index-muscle-loss index quartiles.：NHANES 1999-2004

		**Quartiles of CMI-MLI**			
**Characteristics**	**Overall**	**Q1**	**Q2**	**Q3**	**Q4**
**n**	3185	796	795	797	797
**Age, years**	60 ± 11	58 ± 11	60 ± 12	61 ± 11	61 ± 11
**Sex, n(%)**					
male	1,571 (46%)	402 (44%)	373 (43%)	418 (50%)	378 (48%)
female	1,614 (54%)	394 (56%)	422 (57%)	379 (50%)	419 (52%)
**Marital status, n(%)**					
non-married	1,011 (27%)	259 (26%)	251 (27%)	232 (27%)	269 (30%)
married	2,174 (73%)	537 (74%)	544 (73%)	565 (73%)	528 (70%)
**Education, n(%)**					
elementary school or below	668 (9.3%)	104 (5.9%)	153 (8.7%)	190 (11%)	221 (13%)
secondary school	1,209 (39%)	307 (35%)	277 (36%)	306 (41%)	319 (45%)
college and above	1,308 (52%)	385 (59%)	365 (55%)	301 (48%)	257 (42%)
Race, n(%)					
Non-Hispanic White	1,797 (79%)	451 (78%)	467 (79%)	450 (80%)	429 (78%)
Mexican American	683 (4.3%)	86 (2.5%)	145 (3.6%)	200 (5.0%)	252 (6.5%)
Non-Hispanic Black	503 (9.1%)	227 (15%)	130 (8.7%)	91 (6.7%)	55 (4.5%)
Other Hispanic	113 (4.1%)	15 (2.0%)	29 (4.0%)	34 (5.5%)	35 (5.1%)
Other Race	89 (4.0%)	17 (2.7%)	24 (4.8%)	22 (3.2%)	26 (5.4%)
family PIR, n(%)					
＜1.30	813 (17%)	155 (12%)	185 (17%)	206 (16%)	267 (23%)
1.30-3.50	1,268 (36%)	311 (33%)	309 (34%)	334 (38%)	314 (39%)
＞3.50	1,104 (48%)	330 (55%)	301 (49%)	257 (46%)	216 (39%)
**Smoking, n(%)**					
Never	1,433 (44%)	363 (45%)	373 (47%)	356 (45%)	341 (39%)
Previous	1,193 (37%)	305 (39%)	287 (35%)	301 (35%)	300 (38%)
Current	559 (19%)	128 (16%)	135 (18%)	140 (20%)	156 (23%)
**Drinking, n(%)**					
no	308 (7.9%)	42 (3.8%)	64 (6.4%)	84 (9.6%)	118 (13%)
yes	2,877 (92%)	754 (96%)	731 (94%)	713 (90%)	679 (87%)
**BMI, kg/m^2**	28.5 ± 6.0	25.9 ± 4.9	27.8 ± 5.1	29.8 ± 6.3	31.2 ± 6.2
**WC,cm**	99 ± 15	92 ± 13	97 ± 12	103 ± 14	107 ± 14

**SBP, mmHg**	130 ± 20	128 ± 20	128 ± 20	132 ± 20	132 ± 20
**DBP, mmHg**	73 ± 12	73 ± 11	73 ± 12	73 ± 12	73 ± 13
**FPG, mg/dL**	108 ± 35	100 ± 26	102 ± 21	110 ± 32	122 ± 51
**HbA1c, %**	5.67 ± 0.93	5.46 ± 0.67	5.52 ± 0.62	5.71 ± 0.90	6.05 ± 1.31
**TC, mg/dL**	210 ± 40	204 ± 36	209 ± 35	213 ± 42	217 ± 46
**TG, mg/dL**	154 ± 88	83 ± 25	123 ± 32	164 ± 44	262 ± 105
**HDL-C, mg/dL**	53 ± 16	67 ± 15	56 ± 13	48 ± 11	41 ± 9
**LDL-C, mg/dL**	126 ± 37	120 ± 32	129 ± 32	132 ± 38	124 ± 44
**CRP, mg/dL**	0.48 ± 0.86	0.36 ± 0.87	0.45 ± 0.91	0.53 ± 0.70	0.60 ± 0.89
**Cr, mg/dL**	0.89 ± 0.48	0.89 ± 0.50	0.87 ± 0.26	0.88 ± 0.27	0.91 ± 0.75
**CysC, mg/dL**	0.86 ± 0.37	0.78 ± 0.38	0.83 ± 0.22	0.89 ± 0.25	0.98 ± 0.53
**BUN, mg/dL**	15 ± 6	14 ± 6	15 ± 6	15 ± 6	15 ± 6
**UA, mg/dL**	5.53 ± 1.40	5.06 ± 1.27	5.42 ± 1.34	5.74 ± 1.41	6.01 ± 1.40
**eGFR, mL/min/1.73m^2**	85 ± 19	86 ± 17	85 ± 18	85 ± 19	85 ± 20
**CMI**	2.01 ± 1.65	0.70 ± 0.28	1.31 ± 0.37	2.13 ± 0.57	4.24 ± 1.95
**MLI**	1.02 ± 0.26	0.90 ± 0.20	0.99 ± 0.23	1.05 ± 0.25	1.15 ± 0.30
**CMI-MLI**	0.41 ± 0.78	-0.55 ± 0.32	0.20 ± 0.17	0.74 ± 0.16	1.47 ± 0.33
**MetS, n(%)**	1,478 (43%)	105 (12%)	212 (25%)	473 (57%)	688 (85%)
Hypertension, n(%)	1,906 (53%)	432 (45%)	457 (49%)	488 (56%)	529 (62%)
Hyperlipidemia, n(%)	2,672 (84%)	522 (65%)	615 (79%)	741 (94%)	794 (99%)
DM, n(%)	658 (16%)	95 (8.6%)	105 (9.9%)	201 (19%)	257 (28%)
**CKM syndrome stages, n (%)**					
Stage 0	94 (5.0%)	73 (14%)	18 (3.9%)	3 (0.1%)	0 (0%)
Stage 1	247 (9.6%)	123 (18%)	90 (13%)	26 (4.3%)	8 (1.0%)
Stage 2	1,732 (60%)	397 (52%)	413 (59%)	458 (67%)	464 (65%)
Stage 3	393 (7.4%)	74 (4.7%)	97 (6.8%)	115 (8.6%)	107 (10%)
Stage 4	719 (18%)	129 (11%)	177 (18%)	195 (20%)	218 (23%)
**Advanced CKM stages, n (%)**	1,112 (25%)	203 (16%)	274 (24%)	310 (29%)	325 (34%)

Data were expressed as mean ± SD or numbers (percentages). Participants were categorized into four groups according to the quartiles of baseline cardiometabolic index-muscle-loss index. (Q1 ≤ -0.096, -0.096< Q2 ≤ 0.479, 0.479< Q3 ≤ 1.027, Q4 > 1.027).Percentages were survey-weighted. P values were derived from the Kruskal-Wallis test for continuous variables and the chi-square test for categorical variables.

Abbreviations: Family PIR, Family Poverty–to–Income Ratio; BMI, body mass index; WC, waist circumference; SBP, systolic blood pressure; DBP, diastolic blood pressure; FPG, fasting plasma glucose; HbA1c, glycated hemoglobin; TC, total cholesterol; TG, triglyceride; HDL-C, high-density lipoprotein cholesterol; LDL-C, low-density lipoprotein cholesterol; CRP, C-reactive protein; Cr, creatinine; CysC: Cystatin C; BUN: Blood Urea Nitrogen; UA, Uric Acid; eGFR: estimated Glomerular Filtration Rate; CMI, cardiometabolic index; MLI, muscle-loss index; CMI-MLI, cardiometabolic index-muscle-loss index; DM, diabetes; MetS, metabolic syndrome; CVD, cardiovascular disease; CKM, cardiovascular-kidney-metabolic.

Table S10. Sensitivity analysis: association between CMI-MLI and advanced CKM stages defined solely by G4/G5 CKD (CHARLS).

CMI-MLI	OR (95% CI)	P
Per SD	1.086 (1.008–1.171)	0.031
Q1	ref	—
Q2	0.998 (0.807–1.233)	0.982
Q3	1.003 (0.812–1.241)	0.974
Q4	1.198 (0.971–1.478)	0.092

Adjusted for age, sex, residence place, education, marital status, drinking, smoking, uric acid, blood urea nitrogen, C-reactive protein, diabetes, hypertension, and low-density lipoprotein cholesterol. Advanced CKM stages were defined as Stage 4 plus G4/G5 CKD (eGFR < 30 mL/min/1.73 m²; n = 979).

Table S11. Association between cardiometabolic index-muscle-loss index and advanced cardiovascular-kidney-metabolic stages at baseline: NHANES 1999-2004

	crude model		Model 1		Model 2	
	OR(95%CI)	*P*	OR(95%CI)	*P*	OR(95%CI)	*P*
CMI-MLI per SD	1.49(1.35,1.64)	<0.0001	1.53(1.37,1.70)	<0.0001	1.34(1.19,1.50)	<0.0001
CMI-MLI quartile						
Q1	ref		ref		ref	
Q2	1.75(1.29,2.39)	<0.001	1.61(1.13,2.28)	0.01	1.56(1.09,2.24)	0.02
Q3	2.18(1.66,2.86)	<0.0001	2.06(1.53,2.77)	<0.0001	1.79(1.30,2.46)	0.001
Q4	2.73(2.04,3.65)	<0.0001	2.91(2.14,3.94)	<0.0001	2.15(1.55,2.97)	<0.0001
p for trend		<0.0001		<0.0001		<0.0001

Crude model: unadjusted; Model 1: adjusted for age, sex, family PIR, education, marital status and race; Model 2: adjusted for age, sex, family PIR, education, marital status, race, drinking, smoking, uric acid, blood urea nitrogen, C-reactive protein, diabetes, hypertension and low-density lipoprotein cholesterol. P values were derived from the Wald test.

Abbreviations: CMI-MLI, cardiometabolic index-muscle-loss index; SD, standard deviation; OR, odds ratio; CI, confidence interval.

Table S12. Model comparison for incident cardiovascular disease in the CHARLS cohort.

Model	Terms added	df	AIC	BIC
Covariates only	—	15	23191.79	23270.28

Model	Terms added	df	AIC	BIC
+ CMI	ln(CMI)	16	23182.30	23266.03
+ MLI	ln(MLI)	16	23193.79	23277.51
+ CMI and MLI (separate)	ln(CMI) + ln(MLI)	17	23183.71	23272.67
+ CMI-MLI (composite)	ln(CMI × MLI)	16	23181.78	23265.50

Covariates were age, sex, residence place, education, marital status, drinking, smoking, uric acid, blood urea nitrogen, C-reactive protein, diabetes, hypertension, and low-density lipoprotein cholesterol. Likelihood-ratio test comparing the two-term model with the composite (equal-weight) model: $\chi^2 = 0.068$, df = 1, P = 0.795; comparing the composite with the covariates-only model: $\chi^2 = 12.014$, df = 1, P = 0.0005.

Table S13. Joint associations of CMI and MLI with the risk of incident cardiovascular disease in participants with cardiovascular-kidney-metabolic Stages 0–3.

Group	Adjusted HR (95%CI)	*P* value
Low CMI + Low MLI	1.00 (Ref)	-
High CMI + Low MLI	1.16 (0.99–1.36)	0.0731
Low CMI + High MLI	1.05 (0.89–1.24)	0.5707
High CMI + High MLI	1.23 (1.03–1.46)	0.0195

Model adjusted for age, sex, residence place, education, marital status, drinking, smoking, uric acid, blood urea nitrogen, C-reactive protein, diabetes, hypertension and low-density lipoprotein cholesterol. P values were derived from the Wald test. On the additive scale, RERI = 0.019 (95% CI −0.215 to 0.253); attributable proportion = 1.6%; synergy index = 1.09.

Abbreviations: CI, confidence interval; HR, hazard ratio;; MLI, muscle-loss index; CMI, Cardiometabolic index; CMI-MLI, cardiometabolic index-muscle-loss index.

Table S14. Multiplicative interaction between CMI and MLI on incident cardiovascular disease (CVD).

Interaction term	HR (95% CI)	P value
CMI × MLI (continuous)	1.033 (0.905–1.178)	0.510
CMI_group × MLI_group (median split)	1.009 (0.815–1.249)	0.934

Models were adjusted for age, sex, residence place, education, marital status, drinking, smoking, diabetes, LDL-C, BUN, UA, CRP, and hypertension. Interaction terms were assessed in multivariable Cox proportional hazards models in the CHARLS cohort. P values were derived from the Wald test.

Abbreviations: BMI, body mass index; BUN, blood urea nitrogen; CMI, cardiometabolic index; CRP, C-reactive protein; CVD, cardiovascular disease; HR, hazard ratio; LDL-C, low-density lipoprotein cholesterol; MLI,

muscle-loss index; UA, uric acid.

Table S15. Predictive performance of CMI-MLI and other indices for incident cardiovascular disease in the CHARLS cohort.

Index	AUC (95% CI)	DeLong P value*
CMI-MLI	0.605 (0.588–0.622)	Reference
CMI	0.603 (0.586–0.619)	0.104
MLI	0.600 (0.583–0.617)	0.033
TyG	0.601 (0.584–0.617)	0.026
AIP	0.601 (0.584–0.618)	0.022
TyG-WHtR	0.606 (0.590–0.623)	0.498

*DeLong test comparing each index with CMI-MLI. All models were adjusted for age, sex, residence place, education, marital status, drinking, smoking, diabetes, LDL-C, BUN, UA, CRP, and hypertension.

Abbreviations: AIP, atherogenic index of plasma; AUC, area under the curve; CI, confidence interval; CMI, cardiometabolic index; CMI-MLI, cardiometabolic index–muscle-loss index; MLI, muscle-loss index; TyG, triglyceride-glucose index; TyG-WHtR, triglyceride-glucose–waist-to-height ratio.

Table S16. Association of CMI-MLI with incident CVD within cardiovascular-kidney-metabolic stages 0–3, including participants with outlier values: CHARLS cohort

	Crude model		Model 1		Model 2	
	HR (95% *CI*)	*P*	*HR* (95% *CI*)	*P*	*HR* (95% *CI*)	*P*
CMI-MLI per *SD*	1.15(1.09,1.21)	<0.0001	1.13(1.08,1.19)	<0.0001	1.09(1.03,1.15)	0.002
CMI-MLI quartile						
Q1	ref		ref		ref	
Q2	1.35(1.16,1.58)	<0.001	1.32(1.13,1.55)	<0.001	1.26(1.08,1.48)	0.004
Q3	1.47(1.26,1.71)	<0.0001	1.41(1.21,1.65)	<0.0001	1.28(1.09,1.50)	0.003
Q4	1.56(1.34,1.82)	<0.0001	1.5(1.28,1.75)	<0.0001	1.34(1.14,1.58)	<0.001
P for trend		<0.0001		<0.0001		0.001

Crude model: unadjusted; Model 1: adjusted for age, sex, residence place, education and marital status; Model 2: adjusted for age, sex, residence place, education, marital status, drinking, smoking, uric acid, blood urea nitrogen, C-reactive protein, diabetes, hypertension and low-density lipoprotein cholesterol. P values were derived from the Wald test.

Abbreviations: CMI-MLI, cardiometabolic index-muscle-loss index; CVD, cardiovascular disease; SD, standard deviation; HR, hazard ratio; CI, confidence interval.

Table S17. Association of CMI-MLI with incident CVD within cardiovascular-kidney-metabolic stages 0–3, excluding participants with missing covariate data: CHARLS cohort

	Crude model		Model 1		Model 2	
	HR (95% CI)	P	HR (95% CI)	P	HR (95% CI)	P
CMI-MLI per SD	1.17(1.11,1.23)	<0.0001	1.15(1.09,1.21)	<0.0001	1.11(1.04,1.17)	<0.001
CMI-MLI quartile						
Q1	ref		ref		ref	
Q2	1.35(1.15,1.58)	<0.001	1.32(1.12,1.54)	<0.001	1.26(1.07,1.48)	0.01
Q3	1.44(1.23,1.69)	<0.0001	1.39(1.18,1.63)	<0.0001	1.25(1.07,1.48)	0.01
Q4	1.58(1.36,1.85)	<0.0001	1.51(1.29,1.77)	<0.0001	1.35(1.14,1.59)	<0.001
P for trend		<0.0001		<0.0001		0.001

Crude model: unadjusted; Model 1: adjusted for age, sex, residence place, education and marital status; Model 2: adjusted for age, sex, residence place, education, marital status, drinking, smoking, uric acid, blood urea nitrogen, C-reactive protein, diabetes, hypertension and low-density lipoprotein cholesterol. P values were derived from the Wald test.

Abbreviations: CMI-MLI, cardiometabolic index-muscle-loss index; CVD, cardiovascular disease; SD, standard deviation; HR, hazard ratio; CI, confidence interval.

Table S18. Association of CMI-MLI with incident CVD within cardiovascular-kidney-metabolic stages 0–3, excluding 2020 wave participants: CHARLS 2011–2018

	Crude model		Model 1		Model 2	
	HR (95% CI)	P	HR (95% CI)	P	HR (95% CI)	P
CMI-MLI per SD	1.19(1.12,1.26)	<0.0001	1.16(1.10,1.24)	<0.0001	1.12(1.05,1.19)	<0.001
CMI-MLI quartile						
Q1	ref		ref		ref	
Q2	1.37(1.14,1.64)	<0.001	1.34(1.12,1.60)	0.002	1.28(1.06,1.53)	0.01
Q3	1.39(1.16,1.67)	<0.001	1.34(1.12,1.60)	0.002	1.21(1.00,1.45)	0.05
Q4	1.66(1.39,1.97)	<0.0001	1.57(1.32,1.88)	<0.0001	1.4(1.16,1.68)	<0.001
P for trend		<0.0001		<0.0001		0.002

Crude model: unadjusted; Model 1: adjusted for age, sex, residence place, education and marital status; Model 2: adjusted for age, sex, residence place, education, marital status, drinking, smoking, uric acid, blood urea nitrogen, C-reactive protein, diabetes, hypertension and low-density lipoprotein cholesterol. P values were derived from the Wald test.

Abbreviations: CMI-MLI, cardiometabolic index-muscle-loss index; CVD, cardiovascular disease; SD, standard deviation; HR, hazard ratio; CI, confidence interval.

Table S19. Sensitivity analyses for the association between CMI-MLI and incident cardiovascular disease in the CHARLS cohort.

Analysis	HR (95% CI)	P value
Primary Model 2	1.11 (1.04–1.17)	<0.001
Fine-Gray competing-risk	1.097 (1.040–1.156)	0.00066
Additional adjustment*	1.107 (1.046–1.172)	0.00044
Landmark analysis†	1.088 (1.018–1.163)	0.013

*Additionally adjusted for the frailty index (Fried physical frailty phenotype: robust, prefrail, frail) and hand grip strength. †Excluding CVD events occurring within the first 2 years of follow-up. All models were adjusted for age, sex, residence place, education, marital status, drinking, smoking, diabetes, LDL-C, BUN, UA, CRP, and hypertension. P values were derived from the Wald test.

Abbreviations: CI, confidence interval; CMI-MLI, cardiometabolic index–muscle-loss index; CVD, cardiovascular disease; HR, hazard ratio.

Table S20. Comparison of single-dataset and pooled (Rubin's rules) estimates in the CHARLS cohort.

Outcome	Single-dataset estimate (95% CI)	Pooled estimate (95% CI)
Incident CVD, per-SD HR	1.11 (1.04–1.17)	1.106 (1.045–1.170)
Advanced CKM stages, per-SD OR	2.03 (1.88–2.20)	2.03 (1.88–2.20)

Models were adjusted for age, sex, residence place, education, marital status, drinking, smoking, uric acid, blood urea nitrogen, C-reactive protein, diabetes, hypertension, and low-density lipoprotein cholesterol. Multiple imputation (m = 5) was performed using MICE with predictive mean matching; pooled estimates were obtained using Rubin's rules. HR, hazard ratio; OR, odds ratio; CI, confidence interval; SD, standard deviation; CKM, cardiovascular-kidney-metabolic.
